# How Does Adenine Form from Hydrogen Cyanide?

**DOI:** 10.1021/jacs.5c09522

**Published:** 2026-01-21

**Authors:** Marco Cappelletti, Martin Rahm

**Affiliations:** Department of Chemistry and Chemical Engineering, 11248 Chalmers University of Technology, Gothenburg 412 96, Sweden

## Abstract

The abiotic formation of adenine from hydrogen cyanide (HCN) has long been suspected to be a key step in the origin of life. However, the inherent complexity of HCN’s self-reaction chemistry has challenged researchers for decades, obscuring the detailed mechanistic pathway to adenine. In this study, we employ quantum chemistry and microkinetic modeling to predict and compare four interwoven base-catalyzed pathways to adenine in liquid HCN. Our analysis incorporates both previously proposed aminomalononitrile (AMN) and diaminomaleonitrile (DAMN) intermediates and reveals previously unknown reaction steps, including one in which polyimine can serve as an oxidizing agent. Our modeling offers compelling evidence of a complex, nonequilibrium interplay between these pathways and confirms DAMN as a necessary intermediate. This work establishes a foundational reference for the exploration of abiotic nucleobase formation and highlights how rigorous testing of origin-of-life chemistry pushes the boundaries of state-of-the-art computational chemistry.

## Graphical abstract

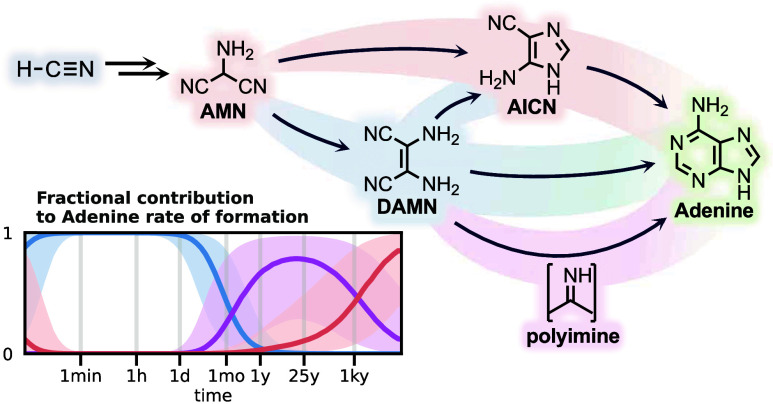

## Introduction

Adenine is present in all life as we know it, where it plays a central role in numerous biochemical processes, including information processing, cellular metabolism, and energy transfer.^1^ The pervasiveness of this nucleobase across biology is a strong indication that adenine has been relevant since the early stages of life’s development.^2^ That this nucleobase was available in the early Earth’s prebiotic inventory is further evidenced by its detection in several carbonaceous chondrites.^3,4^ Despite decades of research, a central question remains: how can this essential building block of life, formally a pentamer of hydrogen cyanide (HCN), form on early Earth and elsewhere? In this work, we use quantum chemistry to evaluate several base-catalyzed reaction mechanisms that can explain the abiotic formation of adenine from HCN. We limit ourselves to reactions in the liquid phase and omit the consideration of radical and photochemically driven processes. Here, we establish pure HCN-based reference mechanisms that emulate anhydrous astrochemical and laboratory conditions while also enabling studies of related transformations in different prebiotically relevant environments. For example, we anticipate this work will facilitate systematic investigations of how water affects adenine formation in cryogenic, ambient, and high temperature environments as well as the potential for mineral catalysis.

The importance of HCN in prebiotic chemistry has been well established since Oró’s groundbreaking detections of adenine from aqueous solutions of HCN and ammonia.^5,6^ Such kinds of experiments do not only yield adenine, but an outstandingly diverse set of compounds, ranging from insoluble black polymers (see, e.g., ref 7), to a wide variety of nucleobases, amino acids, and many other biologically relevant molecules (see e.g., refs 8–10 ). This flurry of complexity emerges from the rich self-reaction chemistry of HCN, which can be initiated by base catalysis or an external source of energy, such as electric discharge, UV light, or other radiation.^7^ The mechanistic details of most such chemistry are not well established.

HCN is ubiquitous in the universe. This small molecule has been repeatedly detected in the interstellar medium, in comets, asteroids, and in various planetary atmospheres.^11–15^ HCN was likely present in Earth’s early atmosphere, oceans, and ponds,^16,17^ albeit at concentrations too low to allow for its polymerization.^18^ Multiple hypotheses have been advanced to explain how HCN may become concentrated enough to allow for self-reactions and drive prebiotic chemistry in such environments. Two prominently debated^19,20^ possibilities include eutectic freezing with water,^21,22^ and HCN generation from formamide.^23,24^

Adenine is currently believed to be (or be close to) the global minimum on the Gibbs energy surface of HCN’s self-reaction chemistry.^25^ In other words, in the chemical landscape of compounds formally composed of H, C, and N in a 1:1:1 stoichiometry, adenine appears to beat all. The large variety of conditions in which adenine can form from HCN is striking and ranges from dilute aqueous solutions^5,21,26^ to anhydrous HCN in both liquid^27–31^ and in the gaseous phase.^32^

While yields of adenine have been reported as high as 22% in experiments where anhydrous HCN reacts with an excess of ammonia at high temperatures,^31^ adenine yields are typically much lower in laboratory experiments (ca 10^–5^ – 10^–1^ %, cf. Table S9). One likely reason for these low yields is statistical, arising from the combinatorial explosion of HCN’s product space,^33^ especially in the presence of water.^34^ However, and as hinted by previous computational investigations,^35–37^ adenine formation is likely also kinetically disfavored over other reaction routes such as, e.g., polyimine.^38–40^ Decades of experiments and computational studies on prebiotic adenine synthesis^37,41–46^ and HCN self-reactions^33,34,40,47–50^ have provided several clues to how adenine is formed. However, because of the experimental challenges associated with characterizing HCN-derived materials,^27,51–55^ the underlying reaction mechanisms are largely unknown. In Scheme 1, we outline previously suggested routes for the onset of HCN’s base-catalyzed self-reactivity, and its proposed subsequent transformation into adenine (**6**).

**Scheme 1:**
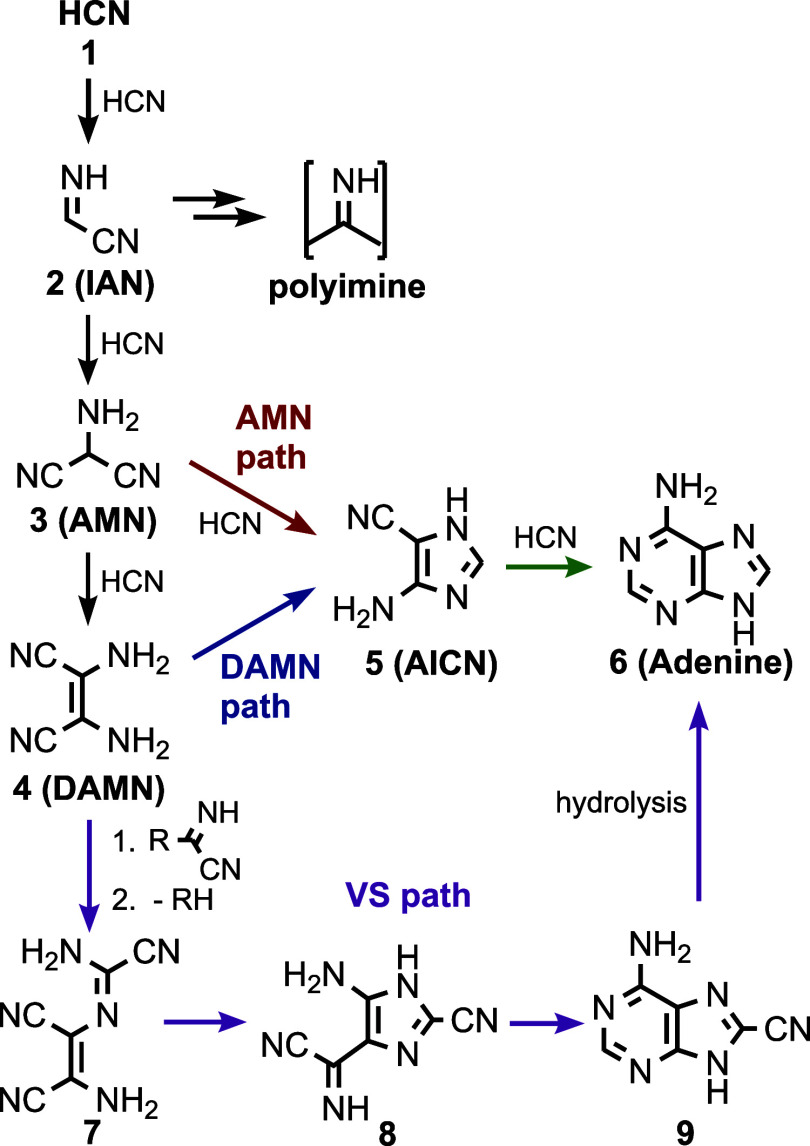
Established^40^ Onset of Base-Catalyzed Self-Reaction Chemistry of HCN (Black Arrows) and Previously Proposed^56–59^ Liquid-Phase Reaction Pathways from HCN to Adenine (Colored Arrows); Arrows Can Represent Multiple Mechanisms; the R Group at the Beginning of the VS Path Was Not Specified But Suggested to Be R = CH_2_NHCN

Both experimental^60,61^ and computational studies^48^ support that the first step of HCN’s self-reaction chemistry is the formation of the transient HCN dimer iminoacetonitrile (IAN, **2**). From **2**, several reaction pathways are, in principle, possible. For example, nucleophilic attack by cyanide anions to the nitrile carbon of **2** can lead to polyimine,^40^ a possibly major component of HCN-derived polymer products^38^ that will play a role in what follows. With near-identical reaction kinetics,^40^ **2** can also transform into the HCN trimer aminomalononitrile (AMN, **3**), which, subsequently, may react to form diaminomaleonitrile (DAMN, **4**).^61^ The HCN tetramer DAMN is a well-established product of HCN polymerization experiments that is distinctly thermodynamically favored.^52,61–63^ Reaction rates,^21,64,65^ thermal analyses,^66^ and the use of DAMN as a reactant,^67^ all suggest that this compound can be an intermediary step toward adenine and other products. However, it has also been suggested that DAMN, partly due to its favorable thermodynamics of formation, may be a dead-end product.^40,61,68^

Both AMN and DAMN have been proposed as plausible reaction intermediates to adenine through different pathways, a selection of which is shown in Scheme 1. Because multiple researchers (see, e.g., refs 57,59,61,64 ) have contributed speculation on the mechanistic details of two of these pathways, we collectively refer to them by the name of their respective starting materials, AMN and DAMN (Scheme 1). Both the AMN and the DAMN pathways have been suggested to converge to 4-amino-1*H*-imidazole-5-carbonitrile (AICN, **5**), an established HCN self-reaction product,^57,64,69^ and one of the first species detected by Oró and Kimball in their early works.^6,69^ AICN is a likely intermediate to adenine, as evidenced by experimental works employing it as a starting reactant.^65,70,71^ The photochemical conversion of DAMN to AICN is well studied, and an additional potential link between DAMN and adenine.^43,65,72,73^ However, because AICN formation has been observed from heated aqueous solutions of NH_4_CN in the absence of UV irradiation,^6,69^ photochemical processes–which are outside the scope of this work–need not be necessary for adenine’s formation. We will, in this work, introduce a revised version of the DAMN pathway that does not proceed through AICN.

A final alternative route to adenine was proposed by Voet and Schwartz (VS, Scheme 1).^58^ This route is partially evidenced by experiments on aqueous HCN and leads to 8-cyano-adenine, **9**, which, in turn, is argued to convert to adenine upon hydrolysis.^58^ This suggestion appears to have been largely ignored until recent experiments with Mg-silicates were found to catalyze HCN oligomerization and resulted in the tentative detection of species along the suggested route.^32^ However, several details of the VS pathway are missing. Most notably, it is not clear how key species **7** may form from DAMN in a first step. Voet and Schwartz have speculated that the imino-tautomer of DAMN, aminoiminosuccinonitrile (AISN, **4′**), may play a role.^58^ The sole indirect evidence for **4′** is observation of glycine,^8^ which could derive from the hydrolysis of the elimination product aminoacetonitrile (RH = NH_2_CH_2_CN, Scheme 1).

Whether adenine is formed through pathways akin to the AMN, DAMN, or VS pathways may depend on the experimental conditions. For example, HCN might in these reactions be substituted for formamide and formamidine, the hydration, and the amination products of HCN, respectively. Formamide has been proposed as an effective HCN-carrier in aqueous (HCN-poor) environments, while formamidine may play such roles and in ammonia-rich environments (see, e.g., refs 8,31,74 ). Hudson et al.’s observations of adenine synthesis from cyanide in liquid formamide^59^ suggest that formamide may promote the AMN pathway. In contrast, the presence of formamidine,^57,65^ or conditions promoting its formation, such as with HCN in liquid ammonia,^29,31^ is consistent with the prevalence of the (conventional) DAMN pathway, where formamidine may help the conversion of DAMN to AICN, and its subsequent transformation to adenine. While the aim of this work is to predict reactivity in pure HCN, the additional complexity that can be brought by considering formamide and formamidine helps emphasize that adenine’s formation may depend significantly on the chemical environment.

Computational methods serve as a crucial complement to experiments and can be particularly suitable for testing hypotheses of HCN’s self-reaction chemistry. Not only is the reactant HCN toxic, its products are complex and challenging to characterize, but experimental evidence for mechanistic details is also rare and often disparate. Computational studies have already shed some light on reactions relevant to the liquid-phase synthesis of adenine. For example, Roy et al.^41^ and Armas-Vázquez et al.^36^ have studied adenine formation from AICN in water. Wang et al. have explored Hudson et al.’s hypothesis by evaluating the pathway from formamide to adenine, via AMN, in formamide and using formamide as a catalyst.^44,75^ While these prior studies relied on implicit solvation models, Sandström and one of us have also used Density Functional Theory (DFT)-based molecular dynamics to study the formation of DAMN from HCN.^40,48^ Despite these efforts, the picture of how HCN can transform to adenine in the liquid phase is far from complete. For example, no computational study has been conducted to test the feasibility of the DAMN pathway in HCN-rich environments. Furthermore, the mechanistic hypothesis by Voet and Schwartz has never been subjected to theoretical scrutiny.

In what follows, we use quantum chemistry to contrast the mechanistic steps of the AMN, DAMN, and VS pathways to adenine in HCN-rich environments. We also provide computational evidence for a different turn of the DAMN pathway that does not necessitate AICN as an intermediate. Furthermore, we detail a plausible scenario for how the unknown first reduction step of the VS pathway may proceed. We finally present the results of microkinetic simulations that reveal the intricate interplay among these different routes to adenine.

## Results and Discussion

A prerequisite for the predictive modeling of reaction pathways is the ability to calculate Gibbs free energy profiles reliably, ideally within 1–2 kcal/mol. In our attempt to approach such accuracy, we rely on a composite quantum chemical protocol outlined in the Computational Methods section, which treats all interacting molecules and a systematically selected set of solvent molecules at a high level of theory, while the surrounding environment is modeled by a homogeneous polarizable continuum. Related methodology has been successful in describing complex metal and enzyme catalysis.^76^ Our calculations model reactions at 278 K (5 °C), a temperature in the middle of HCN’s liquid range at ambient pressure, which matches several previous experiemental^52,77^ and theoretical studies.^40,48^ The concentration of the base catalyst CN^–^ is set to the standard state of 1 M. This choice provides a consistent thermodynamic reference and is broadly representative of reported experimental conditions across solvents (Table S9). Aqueous experiments typically employ stoichiometric amounts of cyanide (pH 9.2, the p*K*_a_ of HCN),^8^ whereas neat HCN conditions span a wider range of concentrations (e.g., 0.5–10% NH_3_, approximately 0.1–3 M of CN^–^).^30,38^ Although we expect our qualitative conclusions to hold near 1 M (SI Section S5), quantitative predictions are sensitive to temperature and the effective cyanide concentration. A systematic exploration of these dependencies is beyond the scope of the present work, and experimental comparisons to our predictions should be made in the context of the specified conditions.

Great strides are being made toward the development of automated reaction discovery codes (see, e.g., ref 78 for a review). Unfortunately, the applications of such protocols to prebiotic chemistry typically sacrifice accuracy for the sake of scale, for example, by relying on semiempirical methods in their initial stages, or by the omission of (explicit) solvation modeling. While we are not critical of such developments, which are both needed and encouraging, we emphasize that care, which often means human intervention, is still needed to reliably predict kinetics and thermodynamics of unknown reaction chemistry in liquid phases.

A major challenge in the modeling of any complex reaction chemistry is conformational sampling. Omission of dynamic sampling of the solvent environment can be motivated but then necessitates careful and chemically sound conformational search. This work relies on a scheme for conformational sampling that applies a combination of several modern stochastic algorithms to all reaction intermediates, transition states (TS), and solvation shells under human supervision. As part of this scheme, we devised a procedure for systematically converging relative Gibbs energies with respect to the solvation shell complexity, i.e., the number of coordinating HCN molecules.

While conformation and solvation effects are important to model complex solution chemistry, our previous work has shown that the quality of the electronic potential often dominates inaccuracies in predicted reaction barrier heights and thermodynamics. For example, it is well-known that generalized gradient approximation (GGA) exchange correlation (XC) DFT, which, for cost reasons, are standard in most molecular dynamics simulations of prebiotic chemistry, can drastically underestimate reaction barriers (by ∼10 of kcal/mol). Even hybrid XC functionals, such as the still popular B3LYP functional, underestimate the activation barriers for cyanide (CN^–^) addition to HCN relative to more accurate ab initio methods (see SI Section S3). In our composite approach, we therefore utilize near-linear-scaling coupled-cluster theory, DLPNO–CCSD­(T)/aug-cc-pVTZ,^79^ to minimize errors in our final relative energy estimates. We mention these technical details at the onset because they are also important when critically evaluating other work on HCN’s reactivity, including routes to adenine, as well as computational reaction chemistry more generally. Taken together, this combination of automated conformational search, cluster-based solvation modeling, and high-level electronic-structure theory allows us to reliably map far more individual reaction steps than has been previously possible, e.g., using DFT-based molecular dynamics studies.^40^

Scheme 2 outlines in more detail the steps of the AMN, DAMN, and VS pathways and our suggested couplings of and additions to these mechanisms. Also indicated is our predicted^40^ route to the formation of polyimine, which we suggest can play a role in adenine formation. Common to all pathways leading to adenine and much of HCN base-catalyzed self-reaction chemistry and polymerization is the formation of IAN and subsequently AMN.

**Scheme 2:**
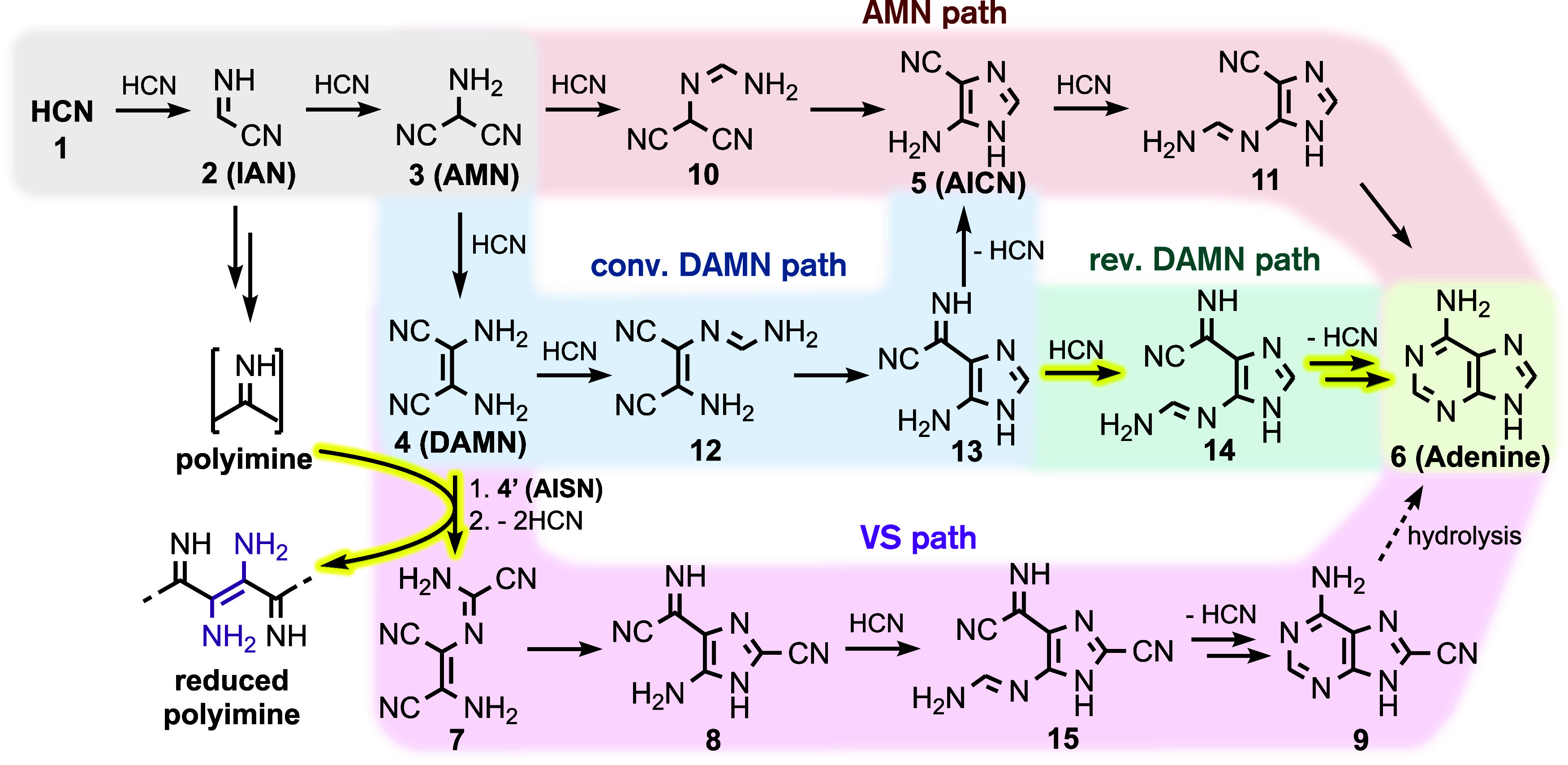
AMN (Red), Conventional (conv.) DAMN (Blue), Revised (rev.) DAMN (Teal), and the VS (Purple) Reaction Pathways to Adenine (Green) in an HCN-Rich Environment^a^

a Mechanistic steps original to this work are indicated by yellow-highlighted arrows. Intermediates between **4** to **7**, from **14** to **6**, and those related to imine ↔ enamine tautomerization are omitted for clarity (they are detailed in Figures S13–S15). Modeling of hydrolysis (dashed arrow) is outside the scope of the current study.

### Beginning: HCN Dimerization and Trimerization

Figure 1 shows the Gibbs energy profile of the first two steps toward adenine, well-studied reactions that serve here as both reference points and method validation. Our best activation energy estimate for HCN’s base-catalyzed dimerization into IAN is 24.0 kcal/mol (**TS1**). This value agrees well with a recent DFT-based molecular dynamics estimate of 21.8 ± 1.2 kcal/mol,^40,48^ but is somewhat higher than the experimental activation energy for aqueous HCN polymerization (∼19.4 kcal/mol).^64^ This difference is consistent with the slower rate observed for the process in the absence of water.^28^ The second step (**TS2**), which results in the trimer AMN (**3**), is predicted to be fast and strongly exergonic, in good agreement with the known transient nature of IAN.^80^

**Figure 1:**
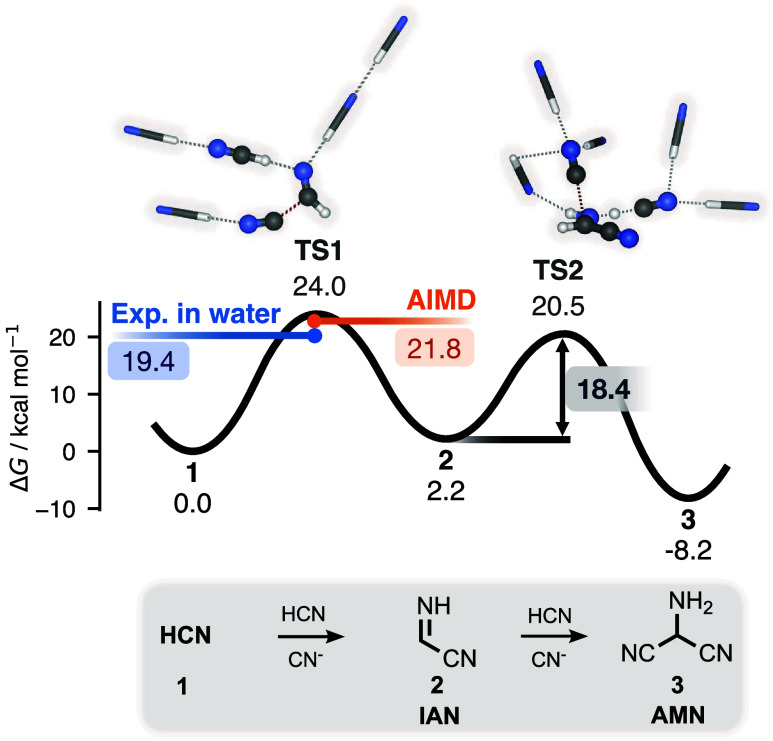
Predicted Gibbs energy profile of AMN formation via IAN. Energies in kcal/mol are provided relative to HCN, except for the **TS2** barrier height, which is shown inside the gray box. DFT-based molecular dynamics results from ref^48^ (in orange), and from aqueous experiments^64^ (in blue) are shown for comparison. Reacting species are shown in ball-and-stick representation; nonreacting solvent molecules are shown as sticks.

One alternative second step omitted from Figure 1 is nucleophilic attack by cyanide on the nitrile group of IAN, for which we compute a barrier of 16.8 kcal/mol from **2**. That second route leads to polyimine, and its close kinetic competition with AMN formation exemplifies a fundamental challenge of modeling prebiotic chemistry: As complexity rises, the number of closely competing, sometimes interlinked, routes grows large. Consequently, it becomes impractical to evaluate the reaction networks in their entirety. This limitation will become increasingly apparent as we progress toward adenine, and we remind the reader that while our study is the most comprehensive to date, it does not rule out determining roles of peripheral (here omitted) chemistry.

### AMN Pathway

The AMN pathway mediated by HCN^10^ is outlined in red in Figure 2. Its first step is **TS5**, which corresponds to an activation barrier of 29.5 kcal/mol, a value that at first glance is consistent with the low observed rate of adenine formation in aqueous phase experiments (Table S9).^8^

**Figure 2:**
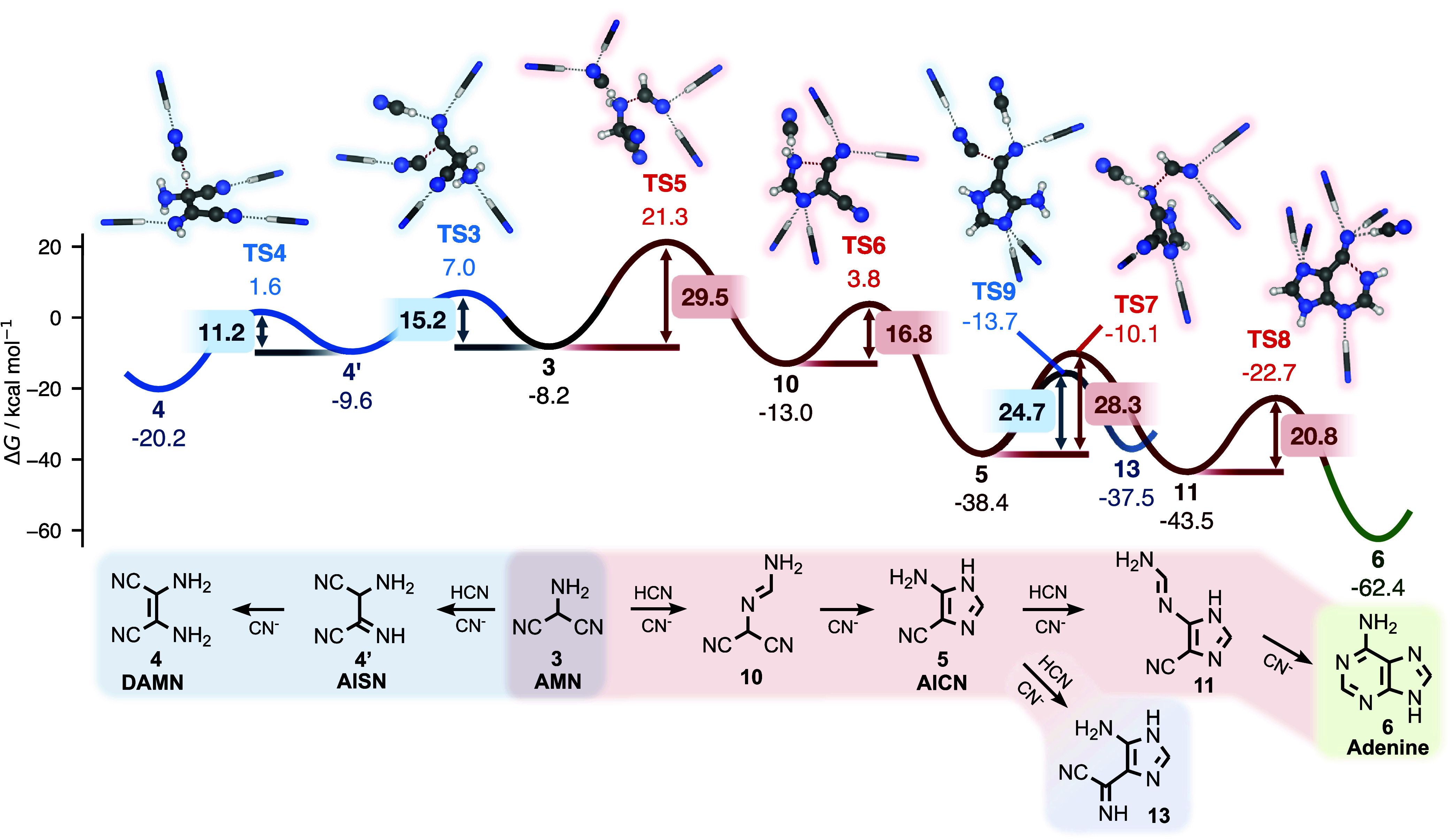
Gibbs energy profile of the AMN pathway shown in red. DAMN formation is also shown for a comparison of kinetics. Intermediates that couple to the DAMN pathway (shown in Figure 3) are highlighted in blue. Energies in kcal/mol are provided relative to HCN, except for selected barrier heights, which are shown inside boxed labels. Reacting species are shown in ball-and-stick representation; nonreacting solvent molecules are shown as sticks.

Notice that already at its onset with AMN (**3**) itself, there is a bifurcation to the DAMN pathway, which we shall return to describe. This fork in the road from the AMN to the DAMN pathways is defined by **TS3**, which lies markedly, ∼14 kcal/mol, below the first transition state of the AMN pathway, **TS5**. Assuming first-order reaction kinetics, this drastic difference in barrier heights translates to approximately 11 orders of magnitude in (forward) reaction rate difference at 278 K. However, this simplistic analysis is illusory: it is not possible to elucidate rate-determining steps from individual barrier heights, as the real rate is determined by the complex interdependence of multiple pathways (a central theme of this work) as well as by the (time-dependent) concentrations of the species involved. For now, it suffices to remember that the AMN pathway is considerably less traversable than previously believed.

For the sake of simplicity, all intermediates following **TS5** are shown in subsequent figures in the enamine tautomeric form. While imines are produced initially in most reaction steps, imine–enamine tautomerization is in all cases fast and strongly shifted to the enamine form. Complete pathways that comprise all intermediates are detailed in SI Section S6.

The remaining bottleneck of the AMN pathway is the nucleophilic addition of AICN (**5**) to HCN via **TS7**, for which we predict a barrier of 28.3 kcal/mol. This step has previously been regarded as strongly kinetically hindered: Roy et al.,^41^ using DFT with two explicit water molecules plus a continuum model, reported an activation enthalpy barrier of 33.9 kcal/mol (at *T* → 0 K), while Armas-Vázquez et al.^36^ found a related transition state with a Gibbs energy barrier of 46.3 kcal/mol. We mainly attribute this latter considerably higher value to the omission of explicitly coordinated solvent molecules.

The subsequent six-membered ring closure via **TS8** is predicted to be comparatively facile, with a barrier of 20.8 kcal/mol. This barrier is highly sensitive to explicit solvation: it decreases by about 7 kcal/mol when the reaction cluster is expanded from one to five HCN solvent molecules (Figure S5). With two explicit HCN molecules, we obtain a barrier of 24.0 kcal/mol, very close to the 24.3 kcal/mol value estimated by Roy et al. with two explicit water molecules. Together, our barriers for **TS7** and **TS8** illustrate how critical it can be to systematically converge reaction and activation energies with respect to the number of explicitly treated solvent molecules in quantum chemistry (SI Section S1.1). Although performing such convergence is more demanding than conventional condensed-phase quantum chemical modeling, it remains far less expensive than DFT-based molecular dynamics, while allowing the use of higher levels of electronic-structure theory.

### Revised and Conventional DAMN Pathways

While DAMN is a stable intermediate in three of the explored four pathways to adenine shown in Scheme 2, we refer to only two of them as “DAMN pathways.” Our revised pathway to adenine is outlined in Figure 3. Formation of DAMN through this route proceeds rapidly from AMN and is followed by a nucleophilic addition reaction through **TS10**.

**Figure 3:**
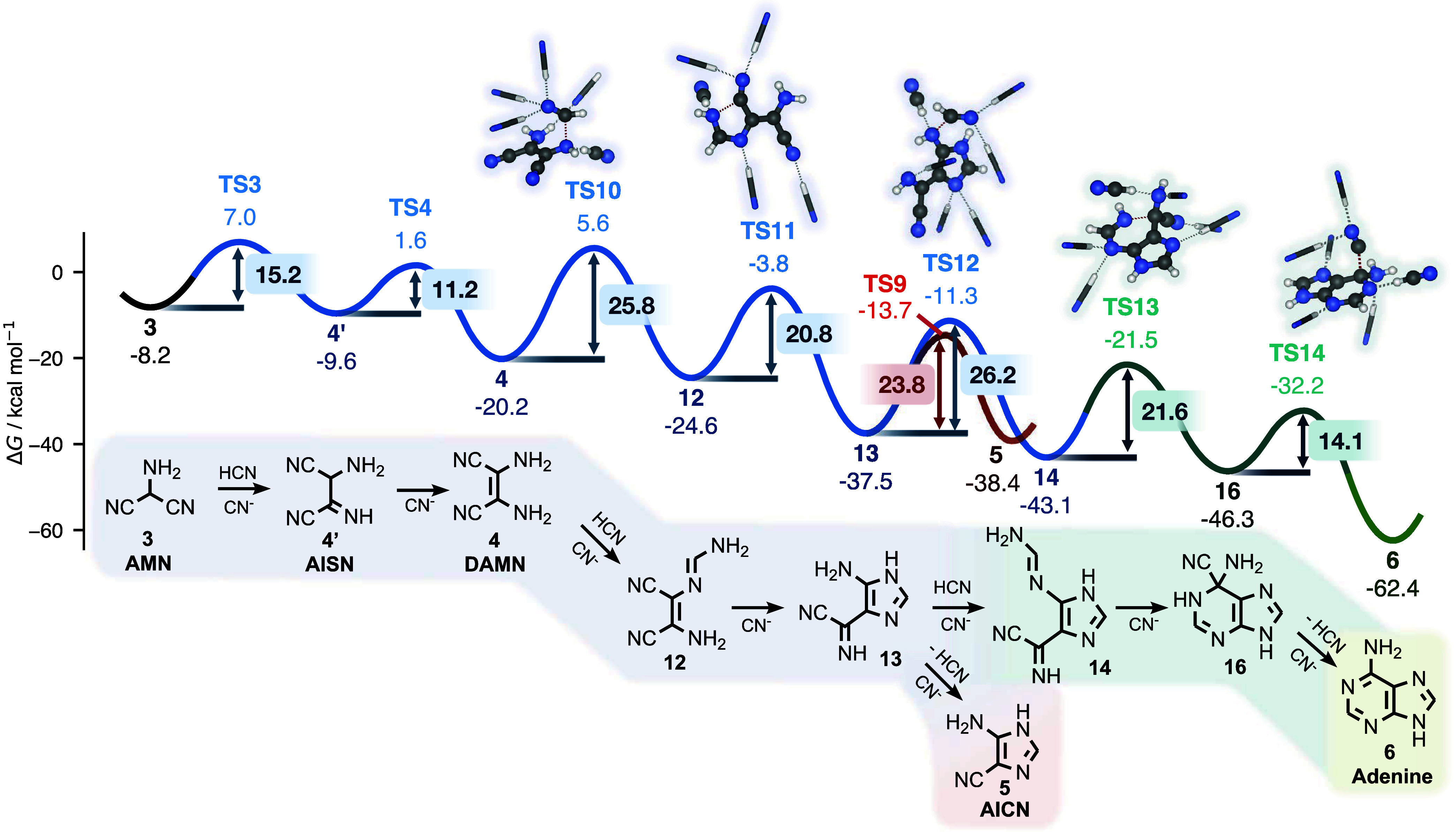
Gibbs energy profile of DAMN pathways, with conventional and newly proposed steps highlighted in blue and teal, respectively. The conventional DAMN pathway connects to the AMN pathway (Figure 2) via AICN, and this coupling is highlighted in red. Energies in kcal/mol are provided relative to HCN, except for selected barrier heights, which are shown inside boxed labels. Reacting species are shown in ball-and-stick representation; nonreacting solvent molecules are shown as sticks.

An important fork in the DAMN road to adenine is intermediate **13**, which forms through a five-member ring closure via **TS11**. In our revised DAMN pathway, **13** transforms to intermediate **14** via **TS12**. In contrast, in the canonical DAMN pathway, species **13** instead connects back to the AMN pathway through **TS9** (blue curve in Figure 2 and red curve in Figure 3) via AICN (**5**).

Which of the two DAMN routes is more kinetically favored? The reaction step to **14** through **TS12** is ∼2.4 kcal/mol higher in energy compared to AICN formation through **TS9**. However, once **14** is formed, the route to adenine through **TS13–14** is considerably more facile than that following the formation of AICN, through **TS7** and **TS8**. The highest barrier of the revised DAMN pathway (**TS12**) is also ∼2 kcal/mol lower compared to that of the canonical route (**TS7**). This suggests a subtle kinetic competition between the two final segments, as we also see from our kinetic modeling.

### VS pathway

Our mechanistic analysis of the reaction hypothesis formulated by Voet and Schwartz^58^ is divided into Figures 4 and 5, and is shown in purple. In Figure 4, we detail the first two steps of our suggested route: the condensation of DAMN and AISN (**4′**) to form **17**, followed by an HCN elimination to give **18**. This part of the mechanism includes its overall rate-determining transition state, **TS15**, with an activation energy of ∼28 kcal/mol from two DAMN molecules.

**Figure 4:**
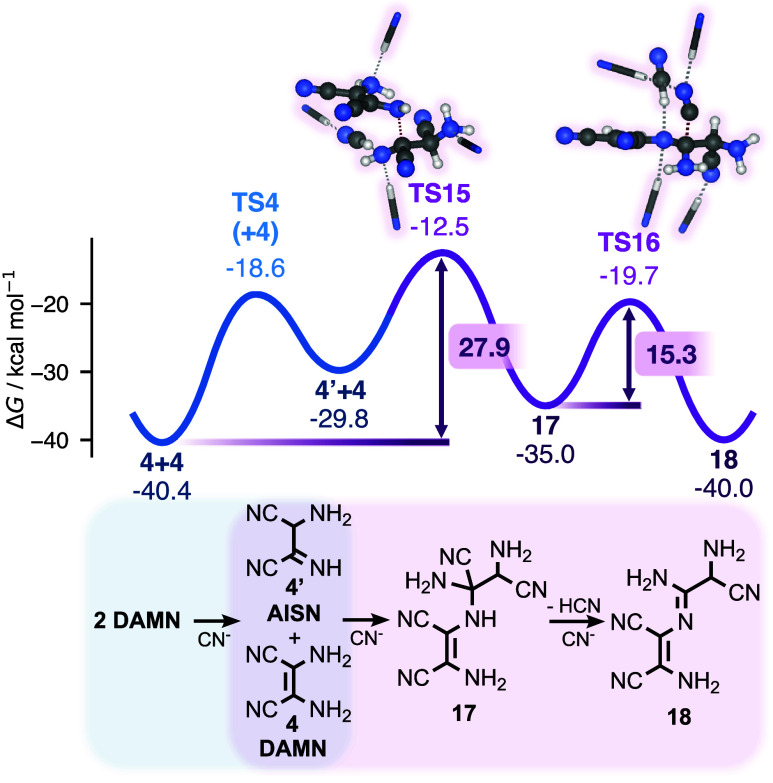
Gibbs free energy profile of the beginning of the VS pathway. Energies in kcal/mol are provided relative to HCN, except for selected barrier heights, which are shown in boxed labels. Reacting species are shown in ball-and-stick representation; nonreacting solvent molecules are shown as sticks.

**Figure 5:**
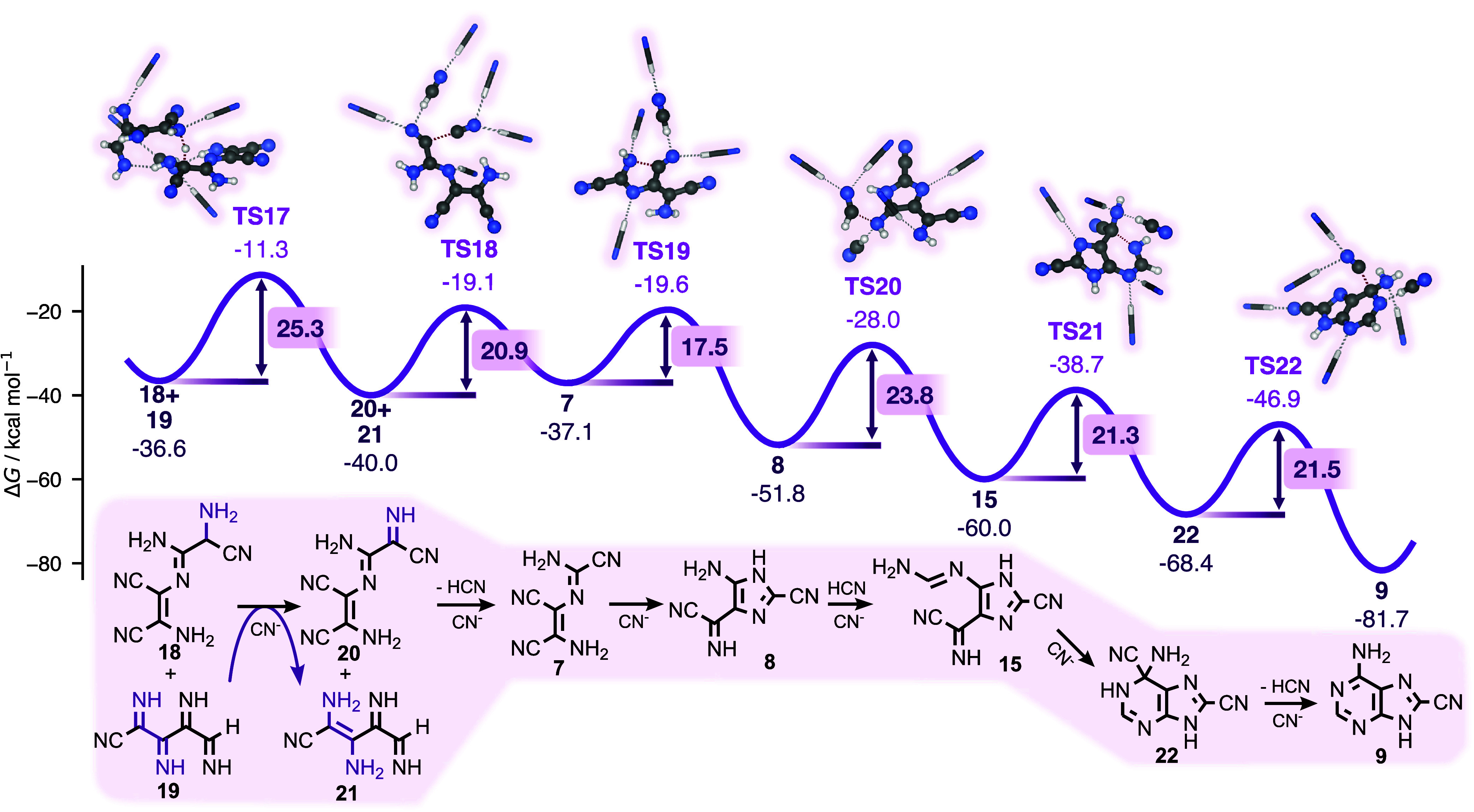
Gibbs free energy profile of the VS pathway, from intermediate **18**. Energies in kcal/mol are provided relative to HCN, except for barrier heights, which are shown in boxed labels. The transformation of the final intermediate **9** to adenine is expected to proceed under suitable hydrolysis conditions but is not explicitly calculated. Reacting species are shown in ball-and-stick representation; nonreacting solvent molecules are shown as sticks.

Despite extensive investigations, we have not been able to identify kinetically feasible mechanisms for the elimination of aminoacetonitrile following condensation of DAMN and AISN, as was originally speculated by Voet and Schwartz. Instead, we suggest an alternative route, in which **18** can react onward to cyano-derivatives via a redox reaction facilitated by polyimine (or a related structure) acting as an oxidizer (Figure 5). In this reaction, a hydride transfer (**TS17**) occurs between **18** and polyimine (represented by a 5 HCN unit fragment, **19**), leading to oxidized intermediate **7** and reduced polyimine fragment, **21**. The presence of polyimine, oligomers or variants of it, is plausible, given its predicted fast formation kinetics,^40^ and the detection of imine groups in pure HCN oligomerization experiments.^38^

Our predicted polyimine-facilitated reaction step **TS17** corresponds to a barrier of ∼25 kcal/mol. We model polyimine as a five-unit HCN oligomer and have confirmed that the barrier height shows no significant dependence on chain length (SI Section S2.3). Nevertheless, this representation remains an approximation of what may proceed in a complex reaction mixture. We note that this (nonconcerted) process resembles the flavin adenine dinucleotide (FAD) redox-active coenzyme reaction, which is able to transform an amino group to an imino group.^81^ Whereas similar reaction mechanisms often require metal catalysts, there is recent precedent for base-catalyzed metal-free hydrogenation with an imine group as hydrogen acceptor.^82^ Moreover, products of redox processes have been observed in HCN self-reaction experiments in the absence of molecular oxygen.^83^ We cannot rule out the possibility that other oxidizing agents produce intermediate **7**. For example, additional species may emerge from HCN’s self-reaction chemistry that comprise two adjacent imino groups or nitrogen-bearing heterocycles (as in the FAD coenzyme). A systematic exploration of such alternative oxidants is outside the scope of this work. Nonetheless, our suggested mechanism is consistent with the claim that HCN oligomers can mediate redox processes.^83^

We envision the VS pathway to proceed to 8-cyano-adenine (**9**) in a manner that is largely analogous to the DAMN pathway (note the similarity between intermediates **7** → **9** with **12** → **6**). The final step from **9** to adenine was postulated by Voet and Schwartz^58^ to proceed quantitative via acid hydrolysis with 8-carboxamide-adenine as an intermediate. We have not studied this final step as it necessitates a different environment than pure HCN. However, we expect it to be feasible under suitable hydrolysis conditions.^84^ In typical HCN polymerization experiments, hydrolysis is carried out under rather harsh conditions: temperatures of 100–140 °C, long reaction times (1–3 days), and use of strong acids (e.g., 6 M HCl; see Table S9). These conditions are compatible with the hydrolysis of **9** to give adenine. For comparison, the time scale (half-life) of nitrile hydrolysis to amide or carboxylic acid in benzonitriles is days to weeks at 85 °C,^85^ whereas cleavage of the carboxamide group in 2-pyridinecarboxamide occurs on the order of hours at 220 °C.^86^ Assuming first-order rate kinetics and using the Eyring equation, these two examples correspond to Gibbs activation barriers of approximately 30–38 kcal/mol under the reported conditions. Strongly catalyzed systems can display substantially lower barriers, in some cases approaching ≤20 kcal/mol.^87–90^

### Dynamical Coupling of Pathways to Adenine

Due to the interwoven nature of the pathways we study, it is not practical to infer a favored mechanism from barrier heights alone. We have therefore performed kinetics simulations of the entire reaction network (Figure 6). Our kinetic model describes neat HCN under basic conditions and, in its baseline form, treats **9** as a dead end of the VS pathway. Where explicitly indicated (e.g., Figure 6c), we also examine scenarios that assume the rapid and quantitative conversion of **9** into adenine, emulating hydrolysis.

**Figure 6:**
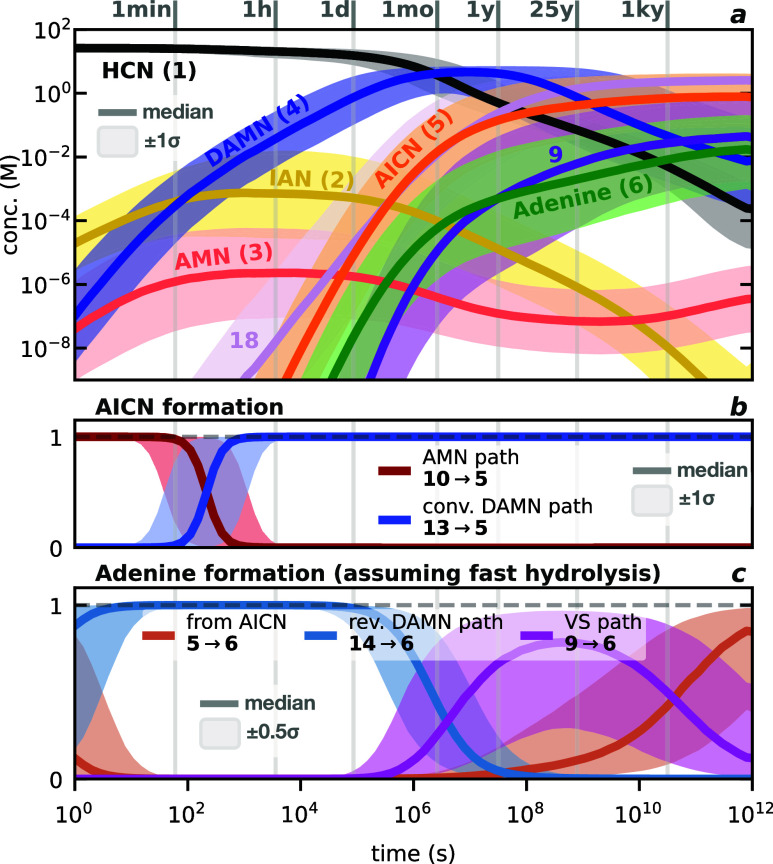
Results of kinetics modeling of all coupled reaction pathways at 278 K. The Monte Carlo approach consists of ∼50,000 simulations, in which both barriers and reaction energies are sampled from a normal distribution centered at our predicted Gibbs energy values, and with σ = 1 (see SI Section S7.2). Colored lines represent medians of distributions. (a) Concentration (log-scale) of selected species with time. Shaded areas are concentration ranges within one standard deviation (±1σ, 68.3%). A detectable ∼nM concentration of adenine is predicted on a time scale of hours. (b) Fractional contribution to AICN rate of formation from **10** (via the AMN pathway, in red) and **13** (via the conventional DAMN pathway, in blue). Shaded areas represent a range within one standard deviation (±1σ, 68.3%). During the first hours of reaction, AICN is mainly formed from AMN. Thereafter, DAMN becomes the predominant intermediate to AICN. (c) Normalized fractional contribution to adenine rate of formation through AICN, **5** (representing both the AMN and the conventional DAMN pathways, orange line), the revised DAMN pathway (via **14**, blue line), and the VS pathway (assuming fast hydrolysis of **9**, purple line). The preferred mechanistic route to adenine is predicted to strongly vary with time. To ease readability due to the large uncertainties, shaded areas here represent 38.3% (±0.5σ) of simulations (see SI Section S7.3).

These simulations entail numerically solving coupled kinetics rate equations at 278 K up to ca. 30 ky (10^12^ s). Such long time scales can be relevant to capture the evolution of chemistry in certain astronomical environments, such as Saturn’s moon Titan. To the best of our knowledge, the longest experiments on HCN polymerization were conducted by Miller and co-workers and ran for 25 years at −20 °C^91^ and 27 years at −78 °C.^22^

Our results shown in Figure 6a concur well with experimental evidence showing (i) that DAMN formation is rapid and (ii) that the equilibrium between AMN and DAMN is strongly shifted toward the latter.^40,52,62^ DAMN quickly becomes the most abundant species in the reaction medium and remains so for up to ∼1 year in the median case.

The colored bands in Figure 6 represent the sensitivity of our full model toward quantum chemical method accuracy and are not to be interpreted as a confidence interval. To obtain these stability estimates, still uncommon in quantum chemistry, we use Monte Carlo sampling that accounts for computational uncertainty in barrier heights and reaction energies (see SI Section S7.2). The bands effectively visualize a best-case scenario, in which we assume that (a) errors in all barriers and reaction energies are uncorrelated and normally distributed, and (b) errors of relative energies are within a standard deviation of 1 kcal/mol. We emphasize that it is not possible to assess the general accuracy and systematic uncertainties of our composite method without large-scale benchmarking of reaction kinetics and thermodynamics. For examples of how individual contributions enter our final Gibbs energy estimates, we refer the readers to SI Section S4.

Our predicted concentration of AMN (**3**, coral line) is well in line with Sanchez et al.’s estimation of ∼10^–5^ – 10^–6^ M from 1 M aqueous HCN, based on kinetic data.^64^ The low concentrations we predict for the dimer IAN (**2**, yellow line) and the trimer AMN are also consistent with an inability to directly detect either in HCN polymerization experiments.

The often-discussed intermediate AICN (**5**, orange line) is predicted to accumulate slowly in the reaction medium, approaching a concentration of ∼0.1 M after months. Figure 6b illustrates that AICN is initially formed through the AMN pathway (from species **10**, red line) on a time scale of minutes, and thereafter, from the conventional DAMN pathway (from species **13**, blue line). This clear shift is enabled by the rapid accumulation of DAMN in the reaction medium, which becomes the key intermediate to adenine.

Adenine itself (**6**, green line), the thermodynamic sink of our reaction network, is predicted to accumulate slowly and become detectable on a time scale of hours. Our median concentration of adenine from the unhydrolyzed medium after 1 month is 10^–4^ M (10^–3^ % of the initial amount of HCN), and 10^–3^ M (10^–2^ %) after 25 years (Table S8). While the heterogeneity of reported reaction conditions precludes a comparison of specific numbers, our results demonstrate clear order-of-magnitude agreement with experimental observations summarized in Table S9. Notably, our predicted 0.3% yield of adenine in the median case at 30 ky (*t* = 10^12^ s) is consistent with Borquez et al.’s experimental estimation of ∼0.1% yield at infinite time from unhydrolyzed HCN-derived products, largely independent of temperature.^92^ In contrast, our value is considerably smaller than the 22% reported by Yamada et al. from HCN in excess liquid ammonia,^31^ a finding that poses mechanistic support for formamidine playing a key role in ammonia-rich environments. The two bottlenecks that we have identified in the conventional DAMN pathway (**TS10** and **TS7**) are precisely the steps that formamidine has been suggested to facilitate.^65,74^

This encouraging agreement between theory and half a century of experiments may still partly reflect fortuitous error cancellation or other limitations of our model. For instance, assuming uncorrelated errors may underestimate systematic uncertainties associated with the chosen electronic-structure method or solvation treatment. Changes in the dielectric constant upon HCN consumption might also alter the interplay between reaction pathways (see SI Section S1.3). Furthermore, the omission of unknown alternative pathways can lead us to underestimate adenine formation, whereas the closed nature of our reaction network may cause an overestimation of the yield on long time scales.

Our modeling of the VS pathway partly addresses the latter concern, as this branch incorporates an intermediate that accumulates over time. Compound **18** (Figure 6a, lilac line), which is involved in the reaction with polyimine (Figure 5), is predicted to be the major species at long time scales. Because **18** is likely reactive, its predicted high concentration at longer time scales is an artifact of the limited reaction network. We therefore consider **18** as a proxy for some part of the 99% of reaction products that do not become adenine. This outcome suggests that it is the redox reaction between **18** and polyimine, via **TS17**, and not DAMN-AISN condensation (via **TS15**), that is the actual bottleneck of the VS pathway. The concentration of polyimine or the presence of other potential oxidizing agents may therefore strongly influence the mechanistic outcome. It is not trivial to provide accurate estimates of the polyimine concentration and formation rates. Side reactions that degrade polyimine or species **18**, and the possibility for polyimine to be reduced multiple times are additional factors not captured by our model. Consequently, because our proposed polyimine-mediated redox step is unprecedented, conclusions that rely on this pathway should be considered provisional.

Within these caveats, our simulations are consistent with Voet and Schwartz’s observations that formation of 8-cyano-adenine (**9**, Figure 6a, purple line) is comparable to that of adenine.^58^ We remark, however, that this qualitative agreement does not by itself establish polyimine as the operative oxidant. If **9** serves as a precursor to adenine under hydrolytic conditions, the VS pathway offers a plausible explanation for the reported increase in adenine yield after hydrolysis.^22,92^ Table S8 summarizes the predicted adenine yields when fast hydrolysis is assumed. In the median case, we find that adenine formation from the VS path becomes significant after 1 year, and by ∼25 years, the majority of adenine originates from **9**. This scenario is consistent with reported adenine yields^22^ from diluted NH_4_CN aqueous solutions stored for 27 years at −78 °C, which increase from ∼10^–4^% to ∼10^–2^% upon acid hydrolysis (Table S9). We note, however, that the predicted formation of **9** carries the largest uncertainty in our network (Figure 6a, purple band), indicating that the VS pathway is more sensitive than others to shifts in the underlying Gibbs energy landscape.

In Figure 6c, we compare the relative contribution to adenine formation over time from AICN (**5**) with that of our revised DAMN pathway (through **14**) and the VS pathway (through **9**), assuming fast hydrolysis. In other words, Figure 6c shows the relative contributions of the AMN and the conventional DAMN pathways combined, as they both proceed through **5**. The main route to adenine is predicted to vary with time.

The AMN pathway, through **10**, is predicted to govern the rate of formation of adenine in the first few seconds. However, at this early stage, the concentration of adenine is arguably too insignificant to be detected in practice. Our revised DAMN pathway (from **4** to **14** and **16**) is predicted to quickly take over and dominate the formation of adenine up to reaction times of months. Thereafter, the VS pathway becomes increasingly important, again if conditions allow for its final hydrolysis step. Finally, at very long time scales, beyond ∼25 years, the canonical DAMN pathway through AICN is expected to become the main route to adenine. This late-time dominance of the canonical DAMN pathway is consistent with the conventional picture for HCN-rich systems,^59^ but the story we see is far more intricate and strongly time-dependent.

The exceptional sensitivity of this chemistry is humbling and serves as a reminder that high accuracy is often needed before meaningful conclusions can be drawn from the simulated dynamics of a reaction network. While we are confident in claiming that multiple pathways compete, the predicted time scales in our analysis are approximate. Nevertheless, our results set the stage for future modeling of the effect of the temperature on the balance between the studied reaction pathways. For instance, will lower temperatures increase the degree of kinetic control, making the AMN pathway further suppressed? Conversely, can higher temperatures increase the relative abundance of AMN relative to that of DAMN, extending the time window in which the AMN pathway dominates?

## Conclusions

We believe this work establishes a foundational reference for understanding the abiotic formation of adenine from HCN, a likely crucial precursor to the origin of life. By quantum chemically modeling base-catalyzed reactions in pure HCN (at 278 K), we, for the first time, compare old and new mechanistic suggestions for how adenine may form on an equal footing. These comparisons effectively couple two canonical reaction hypotheses–through the HCN trimer AMN and the HCN tetramer DAMN–while introducing a competitive new route through intermediate **14**, and proposing several previously missing steps in a largely overlooked pathway suggested by Voet and Schwartz,^58^ which have been tentatively supported by Santalucia et al.^32^ For example, we show that polyimine (a suggested product of HCN polymerization^38^) or related structures can play important roles in initiating the Voet and Schwartz pathway. This route is predicted to account for an appreciable fraction of the total synthesized adenine, provided that hydrolysis of the intermediate 8-cyano-adenine (**9**) is permitted.

Microkinetic modeling reveals that all studied reaction routes share a complex interplay that varies markedly over time. Depending on the time scales considered, different pathways are likely to dominate, prompting a reconsideration of established reaction hypotheses. For example, the canonical DAMN pathway via AICN–long considered dominant in HCN-rich environments^59^–is predicted to take over only at time scales of decades. In contrast, a newly identified mechanism, also based on DAMN but proceeding through intermediate **14**, is predicted to contribute most to adenine formation on shorter time scales.

Our results are consistent with multiple independent observations made over the last half a century. While adenine formation is strongly favored thermodynamically, it is also markedly kinetically hindered; its predicted slow rate of formation is consistent with experiments in pure HCN, where only trace amounts are detected.^27,30^

Our calculations confirm that IAN and AMN are transient species,^64^ and that DAMN forms rapidly after the reaction is initiated and remains as a major intermediate in the reaction medium.^52,62^ It is therefore safe to assume that DAMN likely serves as an intermediate for many other HCN-derived reaction products besides adenine. Our integrated version of the VS pathway supports the claim that redox reactions can take place between HCN-derived products,^83^ providing further insight into the complexity of HCN self-reaction chemistry. This pathway also offers a natural explanation for the well-established increases in adenine yield observed following hydrolysis,^22,92^ provided that suitable hydrolysis conditions are met. While this study of base-catalyzed adenine formation is the most comprehensive to date, our computational screening of reaction mechanisms is necessarily limited. It does not account for side reactions, and given the close competition of the pathways explored, there may well be other closely competing routes to adenine, e.g., from more complex HCN oligomers,^56^ or through other redox reactions.

HCN self-reactivity is likely to play a determining role in much prebiotic chemistry, and its reaction products represent a combinatorial explosion of increasing complexity. Our work, which focuses largely on base-catalyzed reactions in the liquid state, lays the groundwork for future research on prebiotic synthesis of HCN-derived molecules in more complex environments. Understanding which intermediates can persist in detectible quantities should help guide future experiments aimed at exploring the origin of life’s building blocks under different conditions.

Our microkinetic analysis also suggests several concrete, albeit challenging, experimental tests. Long-term polymerization experiments in cold, mildly basic liquid HCN with time-resolved quantification of DAMN, AICN, 8-cyano-adenine, and adenine would directly probe the predicted time-dependent hierarchy of pathways. Such experiments should ideally be run over years and could, for instance, contain added NH_3_ (≈2.4 wt % or ≈4 mol %) to target a nominal cyanide concentration of 1 M. Subsequent systematic variation of hydrolysis conditions (acid strength, temperature, and duration) applied to identical anhydrous reaction mixtures should then help disentangle the role of the VS pathway from other routes. Finally, experiments in which well-defined organic oxidants, for example, suitably chosen hydride acceptors, are added to mildly basic liquid HCN before hydrolysis could test our proposal that redox steps promote the formation of 8-substituted adenine precursors. Answering these questions, and others like them, is ultimately important for reconstructing the chemistry that set the stage for life’s origin.

## Computational Methods

### Molecular Structure

Molecular geometries are optimized at the B3LYP-D3­(BJ)/6–31+G­(d,p) level of theory using Gaussian 16, revision B.01.^93^ The use of B3LYP-D3­(BJ) is motivated by prior studies^40,48^ on HCN that have shown close agreement with interacting HCN experimental geometries.

### Solvation Effects

Solvation effects are modeled by means of a cluster/continuum approach. The solvent association energy, the reaction barrier height, and reaction Gibbs energies are converged within ±1 kcal/mol with respect to the number of explicit HCN solvent molecules, i.e., those that do not partake in the reaction. For consistency, we additionally impose a minimum of four spectating explicit solvent molecules in each cluster for our best estimates (see SI Section S1.1). Each cluster is embedded in Gaussian’s default Polarizable Continuum Model^94,95^ (PCM) for water, with the dielectric constant replaced by that of pure HCN at 278 K, i.e., ε = 144.8.^96^ Section S1.3 shows the sensitivity of the HCN dimerization step with respect to ε.

### Conformational Sampling

Conformational sampling of all solvated clusters is carried out with autodE,^97^ used to generate a set of random noncovalent interacting (NCI) conformers that are subsequently optimized at the DFT level. For steps where the Gibbs activation barrier exceeds 24 kcal/mol, we employ a complementary sampling procedure using the Global Optimizer Algorithm (GOAT)^98^ and the SOLVATOR^99^ algorithm, as implemented in ORCA 6.1,^99^ both at the GFN2-XTB level.^100^ GOAT iterates stochastic uphill pushes and downhill optimization to explore local minima, while SOLVATOR employs the DOCKER algorithm,^99^ based on a form of Particle Swarm optimization, to identify low energy solvation shell geometries. The best candidate identified from all algorithms is then selected. Further details of our conformational sampling workflow are provided in Section S2. All stationary points (minima and transition states) are verified by frequency analysis. Intrinsic reaction coordinate (IRC) calculations are performed for transition states explicitly solvated by a single HCN molecule to confirm the connectivity to the intended intermediates. Visual inspection of the corresponding imaginary-frequency modes in more highly solvated clusters show no meaningful changes. Figure S10 supports this choice by showing no qualitative changes upon explicit solvation.

### Gibbs Energies

Our estimates of the variation of Gibbs energy over reaction steps Δ*G*_A→B_^278K^ in solution are calculated as

1 $${\Delta G}_{A\to B}^{278K}=G_{\left(l\right)}\left(B\right)-G_{\left(l\right)}\left(A\right)$$

where

2 $$G_{\left(l\right)}\left(X\right)=E_{\mathrm{el}}^{\mathrm{CCSD}\left(T\right)}\left(X_{\left(g\right)}\right)+\Delta G_{\mathrm{solv}}\left(X\right)+\Delta G_{\mathrm{th}}^{278K}\left(X_{\left(l\right)}\right)+{\Delta G}_{\mathrm{conc}}^{278K}\left(X\right)$$

*X*_(g)_ and *X*_(l)_ in eq 2 represent a cluster (a molecule and its solvation shell) optimized in the gas phase and with consideration of implicit solvation, respectively. *E*_el_^CCSD(T)^ is the gas-phase total energy calculated with the near-linear-scaling domain-based local pair natural orbital (DLPNO) local correlation approximation to coupled-cluster theory, including single, double, and perturbative triple excitations, CCSD­(T).^79^ The DLPNO–CCSD­(T) calculations are carried out with the correlation-consistent aug-cc-pVTZ basis set and converged below 10^–9^ Hartree, using ORCA 6.1.^99^ Validation of this level of theory against conventional CCSD­(T) is shown in Figure S9. Solvation energies, Δ*G*_solv_, were for all structures except HCN, estimated as,

3 $$\begin{array}{l} \Delta G_{solv}=\Delta G_{solv}^{\mathrm{PCM}}+\Delta E_{relax}\\ =G_{PCM}^{DFT}\left(X_{\left(l\right)}\right)-E_{el}^{DFT}\left(X_{\left(g\right)}\right) \end{array}$$

where *G*_PCM_^DFT^(*X*_(l)_) is the DFT PCM-energy on the *X*_(l)_ geometry, whereas *E*_el_^DFT^(*X*_(g)_) is the DFT gas-phase total energy on the *X*_(l)_ geometry. The Δ*G*_solv_ term thus accounts for the PCM solvation energy (Δ*G*_solv_^PCM^) and the solvent-induced relaxation energy (Δ*E*_relax_).

Δ*G*_solv_(*HCN*) = −2.69 kcal/mol is obtained by the cluster/continuum scheme proposed by Bryantsev et al.,^101^ the details of which are outlined in SI Section S1.2.

Δ*G*_th_^278K^ in eq 2 includes thermal, rotational, and translational enthalpic and entropic corrections to the Gibbs energy evaluated, from the ideal-gas approximation (SI Section S1.4), at the PCM-B3LYP-D3­(BJ)/6–31+G­(d,p) level of theory. For these estimates, small frequencies (<100 cm^–1^) are treated with the rigid-rotor-harmonic-oscillator (RRHO) approximation.^102^

Δ*G*_conc_^278K^ ensures the correct standard-state concentration as,

4 $$\Delta G_{\mathrm{conc}}^{278K}=\mathrm{RT}\ln\frac{c_{\left(l\right)}^{o}}{c_{\left(g\right)}^{o}}$$

where *c*_(l)_^o^ and *c*_(g)_^o^ are the standard-state concentrations in the liquid phase (26.05 M for HCN, 1 M for all of the other species) and gas phase (1 atm), respectively. The relevant concentration corrections are Δ*G*_1 atm→1M_^278K^ = 1.73 kcal/mol and Δ*G*_1 atm→26.05M_^278K^ = 3.53 kcal/mol (the density of HCN is 0.704 g/cm^3^ at 278 K).^96^

### Microkinetic Modeling

The concentrations of all species are resolved in time by numerically solving the set of differential rate equations of the reaction network (a Python script is provided alongside the SI). We set the initial conditions for these simulations to *T* = 278 K, and a concentration of zero for all species except HCN, whose pure liquid concentration is 26.05 M. Kinetics constants are derived from the Eyring equation

5 $$k=\frac{k_{B}T}{h}\exp\left[-\frac{\Delta G^{‡}}{\mathrm{RT}}\right]$$

where *k*_B_ is the Boltzmann constant, *T* is the temperature, *h* is the Planck’s constant, *R* is the gas constant, and Δ*G* ^‡^ is the barrier height. Reaction rate constants are evaluated from Gibbs energies corrected to a 1 M standard state for all species, including HCN.

Kinetically irrelevant steps, e.g., proton transfers and tautomerizations, are omitted. For the formation of polyimine, a self-initiated step-growth polymerization is assumed, for which we compute a reaction Gibbs energy of +0.95 kcal/mol (SI Section S7.4).

Uncertainties of concentrations are estimated using a Monte Carlo approach, in which all barrier and reaction energies are varied independently and stochastically. Energies are sampled quasirandomly from normal distributions centered around our quantum chemical estimates shown in Figures 1–5, with a standard deviation of 1 kcal/mol. An ensemble of ∼50,000 simulations is used to obtain the median and the 0.159 and 0.841 quantiles, corresponding to the 68.3% (1σ) interval of the concentrations. Further details of the kinetics modeling are provided in SI Section S7. SI Table S8 presents the computed adenine yields and their comparisons with experimental data.

## Supplementary Material



## Notes

Solvation modeling, conformational search, validation of DLPNO–CCSD­(T), analysis of contributions to relative Gibbs energy estimates, proton transfer and tautomerization, microkinetic modeling, link to data set with Gaussian 16 and ORCA 6.1 input and output files, and Python script for microkinetic modeling (PDF)
