# Supplementary material for: How Does Adenine Form from Hydrogen Cyanide?
Source: J Am Chem Soc. 2026 Jan 21;148(4):3949–61. doi: 10.1021/jacs.5c09522 (PMC12879745; doi:10.1021/jacs.5c09522)
Supplement: Supplementary file 1 [file ja5c09522_si_001.pdf]

Supporting Information for

How Does Adenine Form from Hydrogen Cyanide?

Marco Cappelletti[1], Martin Rahm[1,*]

[1]Department of Chemistry and Chemical Engineering, Chalmers University of Technology, Gothenburg 412 96, Sweden

*Corresponding author: martin.rahm@chalmers.se

Table of contents

1. Solvation Modeling 3

1.1 Explicit Solvation 3

1.2 The Solvation Energy of HCN 7

1.3 Sensitivity with respect to dielectric constant 9

1.4 Evaluation of entropic changes 9

2. Conformational Search 10

2.1 Default conformational sampling scheme 10

2.2 Enhanced conformational sampling for key reaction steps 11

2.3 Validating modeling of polyimine-facilitated redox reaction step 11

3. Validation of DLPNO-CCSD(T) 11

4. Analysis of Contributions to Relative Gibbs Energy Estimates 12

5. Sensitivity to Cyanide Concentration 13

6. Proton Transfer and Tautomerization 13

7. Microkinetic Modeling 18

7.1 Kinetics Equations 18

7.2 Monte Carlo Sampling of Reaction Parameters 19

7.3 Contributions to the Rate of Formation of AICN and Adenine 21

7.4 Polymerization of Polyimine 22

7.5 Adenine yield against experiments 22

8. References 24

Dataset

The dataset containing optimized structures, Gaussian16 and ORCA 6.1 input and output files, as well as script and results of the microkinetic modeling are available at the Swedish National Data (SND) Service:

https://doi.org/10.71870/gqyb-fj64

1. Solvation Modeling

1.1 Explicit Solvation

In our cluster model of solvation each reaction complex is both implicitly solvated using the Polarizable Continuum Model (PCM) while also being coordinated to several HCN molecules that do not partake in the reaction. Explicit solvation modeling must be handled with care, especially when predicting reaction barriers, as non-cancelling errors may otherwise arise [1]. In this work, we encounter reactions in which the change in HCN molecules from the reactant to the product state, *Δ(HCN)*, can be either of three:

- HCN additions: *Δ(HCN) = +1*
- HCN eliminations: *Δ(HCN) = -1*
- Intra-molecular cyclization reactions, proton transfers, or hydride transfers: *Δ(HCN) = 0*

To this end, we consistently incorporate a constant number of non-reacting explicit solvent molecules throughout each reaction step, which minimizes the risk of artificial shifts in the relative Gibbs energy due to an unequal solvation description. How the number and disposition of explicit solvent molecules is decided is described in the next paragraph. Figure S1 serves to exemplify an HCN addition, *Δ(HCN) = +1*. In the example reaction step of Figure S1, one HCN solvent molecule is added to the reaction, i.e., it is being removed from the solvent bath. How we model the energy of HCN dissolved in HCN is described in Section S1.2. Explicitly coordinated HCN solvent molecules are in this example depicted as sticks, while the addition of HCN molecules from the solvent state are indicated by "+ HCN". Note that the added solvent molecule in this instance is not added to the HCN dimer iminoacetonitrile (IAN, **2**), but to the structure **TS2**, which precedes the formation of aminomalononitrile (AMN, **3**). This choice is made because the solvent molecule coordinated to the imine group in **2** is spent regenerating the base CN^- catalyst in **TS2**. In other words, by coordinating one additional solvent molecule to the reacting CN^- anion in **TS2,** we compensate for this removal. Where in the molecule the added solvent molecule is coordinated is determined by energy minimization coupled to unbiased conformational sampling, as described in Section S2.

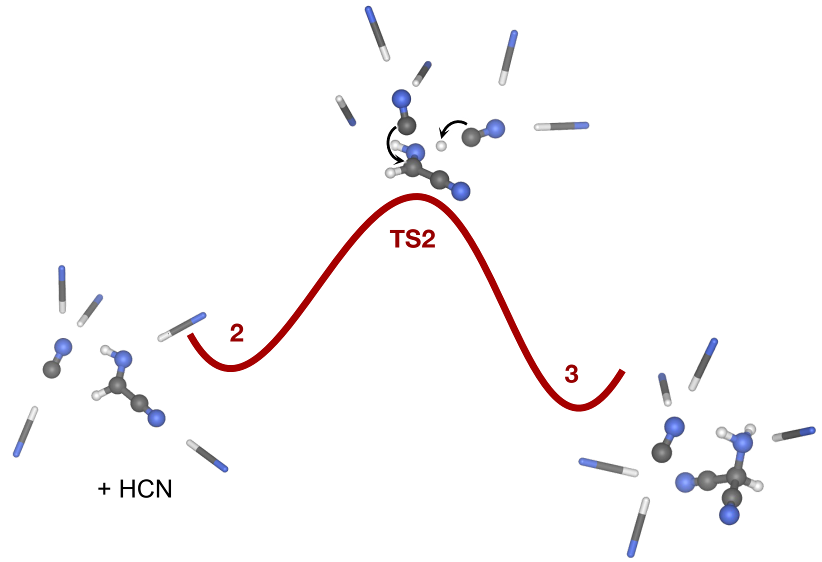

Figure S1: Example of Gibbs energy profile of an HCN addition reaction, showing the computed clusters, where five explicit solvent molecules (shown as sticks) are considered. "+ HCN" indicates an HCN solvent molecule that is not coordinated to the reaction complex, but adds to it during the transition state. The number of non-reacting solvent molecules are constant over the reaction step.

In this work, we use a cluster approach in which (i) the solvent association energy, i.e., the change in Gibbs energy ΔG(HCN) due to transferring one HCN molecule from the solvent to the reaction cluster or transitions state, (ii) the reaction barrier height $\Delta\Delta G^{\ddagger}$, and (iii) reaction energies $\Delta\Delta_r G$, are all three converged within ±1

kcal/mol with respect to the number of non-reacting HCN molecules in the cluster. Our tests show that when these criteria are combined, results are robust. While most reaction steps studied in this work meet these convergence criteria at four units of HCN or below, a small subset required five solvent molecules in the cluster. For our final data set, we at minimum use four explicit non-reacting solvent molecules.

Figure S2 shows convergence of ΔG(HCN) (one of the three criteria) for four complexes: species **15** and the six-member ring formation from it **TS21**, DAMN (**4**), and its nucleophilic attack to HCN, **TS5**. For these structures the cluster models all converge at four explicit HCN solvent molecules.

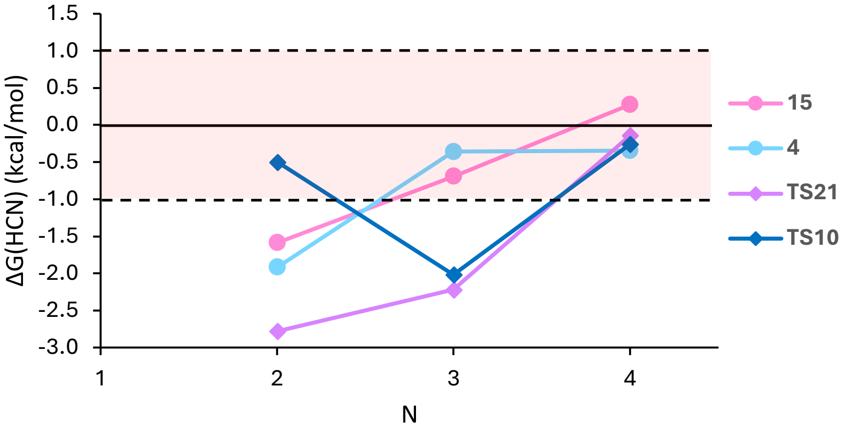

Figure S2: Change in Gibbs energy (in kcal/mol) upon associating one additional solvating HCN, ΔG(HCN), as a function of the resulting number of explicit HCN solvent molecules, N, for four selected species. The shaded red area represents the convergence threshold (±1 kcal/mol).

Figure S3 shows a complete convergence example for the HCN dimerization reaction (**1**→**2** via **TS1**), illustrating how results vary when transitioning from no explicit solvation (leftmost barrier height) to four explicit HCN molecules (rightmost barrier height). Figures S4-S7 show the evolution of all barrier heights in our reaction network against the number of explicit HCN molecules. Notably, most (not all) barrier heights decrease with increased solvation, an effect that is particularly pronounced for cyclization reactions (depicted as triangles in Figure S5-S7).

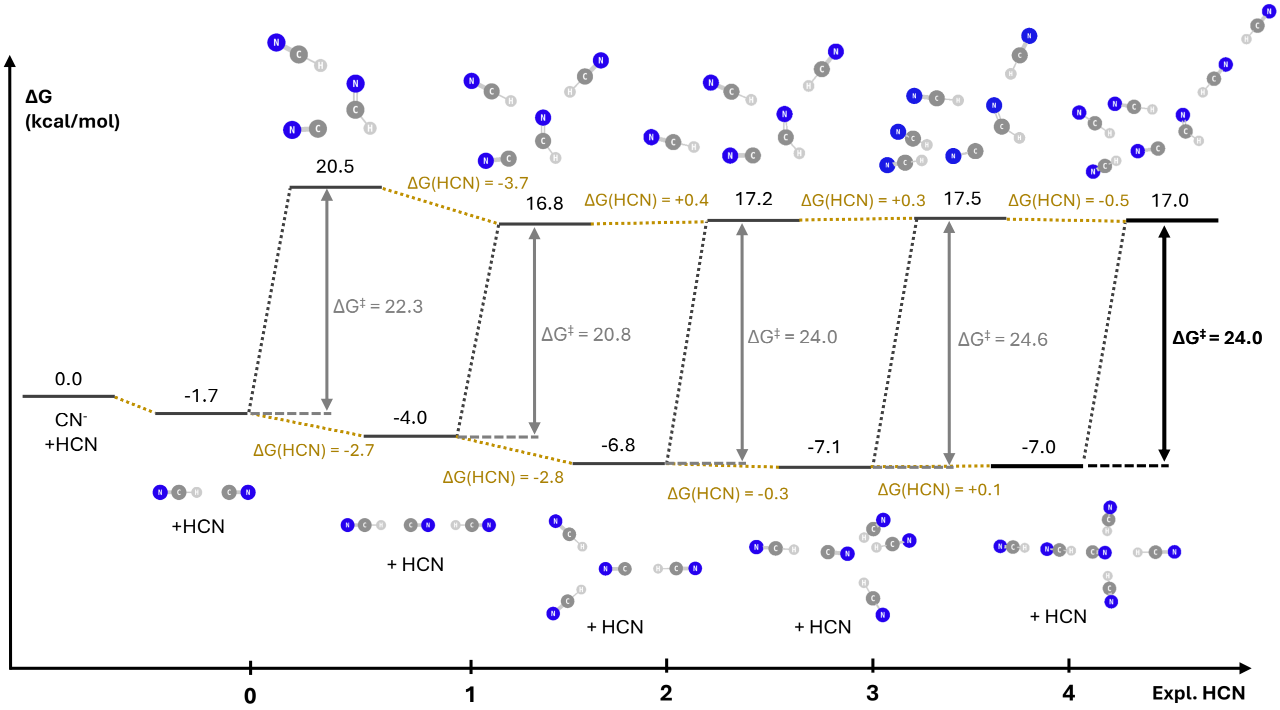

Figure S3 Effect of including multiple HCN solvent molecules in PCM modeling of the HCN dimerization reaction (**1** → **2** through **TS1**). "+ HCN" indicates an HCN molecule in the solvent state. In this example, both the solvent association energy, the barrier height, and the reaction energy are converged below 1 kcal/mol already at N=3. We have nonetheless used a minimum of N=4 in our best estimate for all reaction steps.

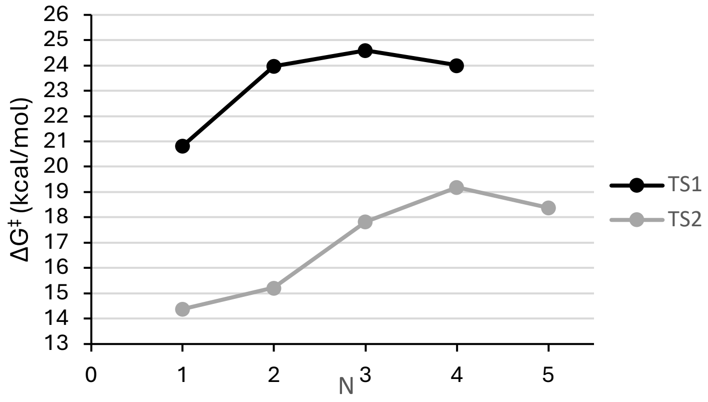

Figure S4: Convergence of barrier heights with respect to the number of explicit HCN solvent molecules (N) for the beginning of HCN self-reaction (**1** to **3**, cf. Figure 1).

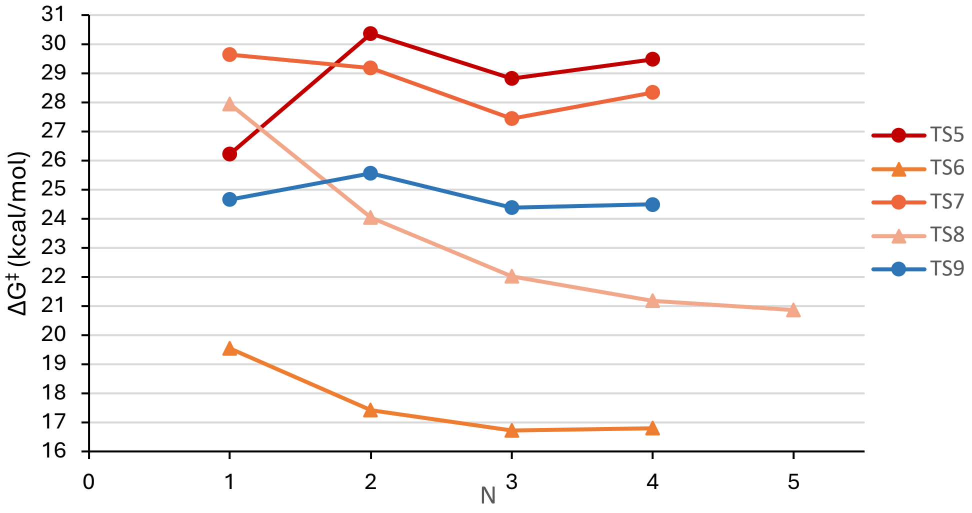

Figure S5: Convergence of barrier heights with respect to the number of explicit HCN solvent molecules (N) for the AMN pathway (cf. Figure 2). Cyclization reactions are marked with a triangle.

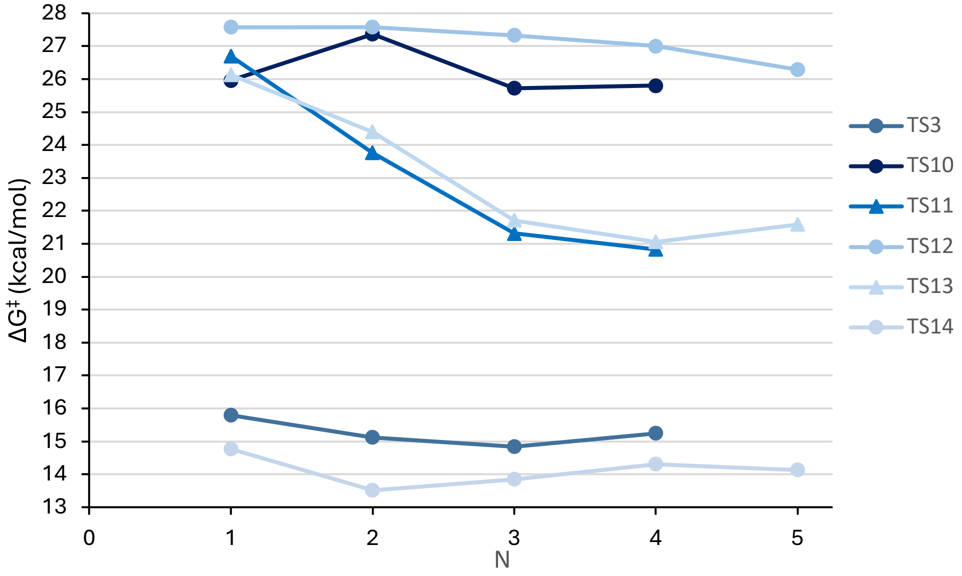

Figure S6: Convergence of barrier heights with respect to the number of explicit HCN solvent molecules (N) for the DAMN pathway (cf. Figure 3). Cyclization reactions are marked with a triangle.

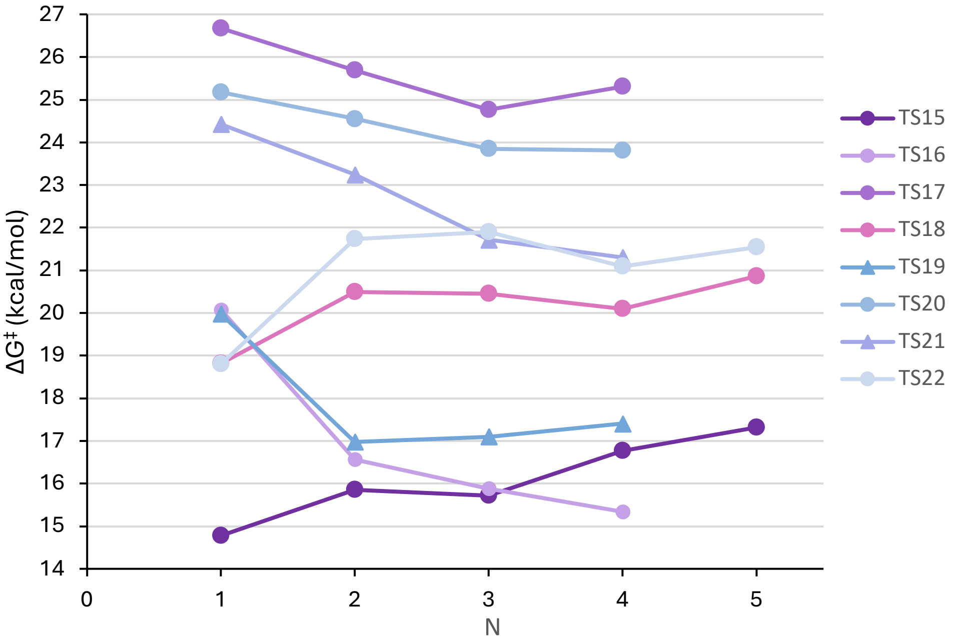

Figure S7: Convergence of barrier heights with respect to the number of explicit HCN solvent molecules (N) for the Voet and Schwartz's pathway (cf. Figures 4-5). Cyclization reactions are marked with a triangle.

1.2 The Solvation Energy of HCN

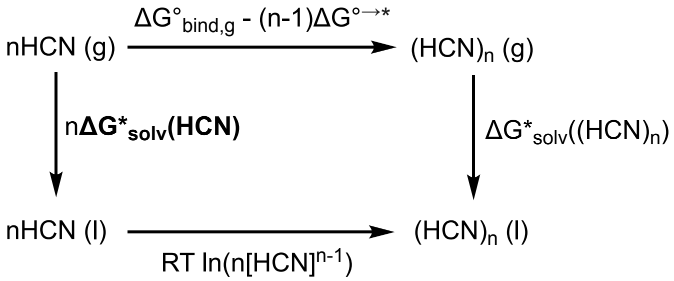

Scheme S1: Thermodynamic cycle of HCN in gas and liquid phase [2]. Superscript asterisk (*) represents standard state conditions in solution ($[HCN]_{(l)}$ = 26.05 M), while superscript circle (°) represents the standard state conditions in gas phase (1 atm).

The solvation energy of HCN in itself, $\Delta G^*_{solv}(HCN)$, has been estimated by means of the cluster/continuum scheme proposed by Bryantsev et al. [2]. Given the high computational costs, this scheme proved feasible only for HCN. In the Bryantsev et al. approach, $\Delta G^*_{solv}(HCN)$ is obtained from the thermodynamic cycle shown in Scheme S1 [2], following computation of $(HCN)_n$ clusters with increasing size n, both in vacuum and in implicit solvation, and extrapolation to the limit for $n \to \infty$ (Figure S8). In Bryantsev et al.'s notation, the superscript asterisk (*) represents standard state conditions in solution, while the superscript circle (°) represents the standard state conditions in gas phase (1 atm). $\Delta G^{\circ \to *}$ stands for the change in Gibbs energy from the gas phase to the liquid phase, which equals 1.73 kcal/mol at 278 K. The solvation free energy was extrapolated to the $n \to \infty$ limit from $(HCN)_n$ clusters with n = *2, 3, 4, 5, 6*, using the equation:

$$\Delta G^*_{solv}(HCN, n) = \Delta G^*_{solv}(HCN) + ae^{-bn}. \quad \text{(S1)}$$

The global minimum of $(HCN)_n$ clusters is predicted to be linear up to $n = 4$, while a planar cyclic conformation is favorable for larger sizes, in accord with previous reports [3], [4]. These predictions are supported by sampling of $(HCN)_n$ clusters (up to $n = 6$), using the Conformer-Rotamer Ensemble Sampling Tool (CREST) [5], combined

with the semiempirical quantum mechanical method GFN2-xTB [6], followed by optimization of lowest energy structures with PCM-B3LYP-D3BJ/6-31+G(d,p) in Gaussian 16 [7].

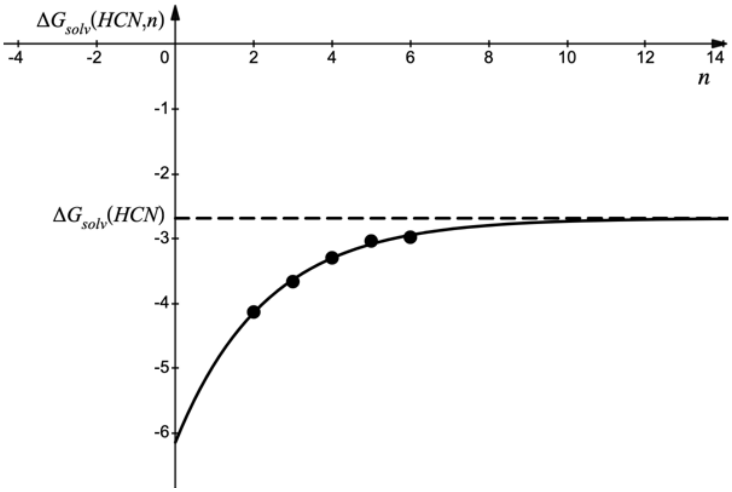

Figure S8: Computed values of $\Delta G^*_{solv}(HCN, n)$ with n = 2, 3, 4, 5, 6, and the extrapolation to $\Delta G^*_{solv}(HCN)$, as in equation (S1). Energy refinement with DLPNO-CCSD(T)/aug-cc-pVTZ.

The Bryantsev et al.'s cluster/continuum scheme provides a solvation Gibbs energy only 0.8 kcal/mol from experimental estimates of -3.52 kcal/mol (Table S1). Table S1 includes validation of $\Delta G^*_{solv}(HCN)$ calculated with DLPNO-CCSD(T) against conventional CCSD(T), which agree closely.

Table S1: Gibbs energy for solvating HCN in HCN, $\Delta G^*_{solv}(HCN)$, in kcal/mol). [a]Experimental value provided as RT ln([$HCN_{(g)}$]/[$HCN_{(l)}$]), where [$HCN_{(g)}$] is the saturation vapor density derived from Ref. [8], and [$HCN_{(l)}$] = 26.05 M. See Ref. [2].

**Method for energy refinement**	$\boldsymbol{\Delta G^*_{solv}(HCN)}$
DLPNO-CCSD(T)/aug-cc-pVTZ	-2.69
CCSD(T)/aug-cc-pVTZ	-2.71
**Exp.**[a]	-3.52

1.3 Sensitivity with respect to dielectric constant

We here evaluate the sensitivity of our results with respect to the dielectric constant ε used as a parameter in the implicit solvation modeling using PCM. This comparison is relevant for our system since ε may in principle change during polymerization as HCN is consumed to form self-reaction products. Table S2 shows the effect of modifying ε on the barrier and reaction energy of HCN dimerization (**1** → **2** via **TS1**), including a single solvent HCN molecule. Our model (ε = 144.8) is compared with the PCM default for water at 298.15 K (ε = 78.35). We did not apply Bryantsev et al.'s scheme for the water case. Overall, changing ε has but a minor effect on the kinetics and thermodynamics of the first step.

Table S2: Effect of modifying the dielectric constant (ε) in our PCM treatment of the HCN dimerization reaction (**1** → **2** via **TS1**) kinetics and thermodynamics. Gibbs free energies are reported in kcal/mol, referenced to species **1**.

PCM dielectric constant	TS1	2
ε = 144.8 (HCN at 278 K)	20.8	1.5
ε = 77.35 (H_2O at 298.15 K)	20.5	1.2

1.4 Evaluation of entropic changes

Entropic corrections from static electronic-state calculations are evaluated by treating the translational, rotational, vibrational, and electronic degrees of freedom as independent, each obtained from the corresponding single-particle partition function. These partition functions are derived within the ideal-gas approximation, assuming harmonic modes and neglecting contributions from excited electronic states. Furthermore, implicit solvation models such as PCM are parametrized to reproduce experimental Gibbs energies of solvation, meaning that the individual enthalpic and entropic terms are not strictly physical [9]. Together, these factors contribute to an uncertainty of Gibbs energy corrections.

To assess the reliability of the PCM model in providing accurate Gibbs energies of solvation, we compared our results with Wertz's approach for estimating solvation entropies [10]. In this empirical but physically motivated method, the entropy of solvation is estimated by assuming that each solute loses a fixed fraction of its gas-phase entropy. This assumption is based on the observation that most entropy loss upon solvation arises from restrictions in translational and rotational degrees of freedom. In Wertz's approach, the solvation entropy of a species X, $\Delta S_{solv,W}(X)$, is given by:

$$\Delta S_{solv,W}(X) = -f\left(S°_m\left(X_{(g)}\right) - \Delta S^{\circ\to *}\right). \tag{S2}$$

Where f is the fraction of entropy lost upon transfer from the gas phase to solution, $S°_m\left(X_{(g)}\right)$ is the standard molar entropy of X in the gas-phase, and $\Delta S^{\circ\to *}$ is the entropy change associated with passing from the gas phase ($p°$ = 1 atm = 0.04 M) to the liquid phase ($p*$ = 55.5 M for water), i.e.:

$$\Delta S^{\circ\to *} = R \ln\left(\frac{p^*}{p^\circ}\right). \tag{S3}$$

For liquid HCN at 278 K ($p*$ = 26.05 M), this corresponds to $\Delta S^{\circ\to *}$ = 12.7 cal mol^{-1} K^{-1}. Furthermore, given $S°_m\left(HCN_{(g)}\right)$ = 48.2 cal mol^{-1} K^{-1} [11] and $S°_m\left(HCN_{(l)}\right)$ = 27.0 cal mol^{-1} K^{-1} [12], we find f = 0.24.

When accounting for Wertz's solvation entropy, the Gibbs energy of a species X, $G_W^{278K}(X)$, is then obtained as:

$$G_W^{278K}(X) = \Delta H_{th}^{278K}\left(X_{(l)}\right) - T\left(\Delta S_{th}^{278K}\left(X_{(l)}\right) + \Delta S_{solv,W}(X)\right) \tag{S4}$$

Where ΔH_{th}^{278K} and ΔS_{th}^{278K} are the enthalpy and entropy thermal corrections, respectively. In Table S3 we compare the results of three reactions with different molecularity m: via **TS2** (m = -1), **TS6** (m = 0), and **TS9** (m = +1).

To lower computational requirements for this comparison, we consider clusters including a single explicit HCN solvent molecule (as opposed to a minimum of four in our main dataset). Tables S3 shows that the two methods agree within 1 kcal/mol, supporting the use of the ideal-gas entropic correction for our purposes.

Table S3: Comparison of relative Gibbs free energies (kcal/mol) calculated with our approach (**$\Delta G^{\ddagger}$** and **$\Delta_r G$**) and including Wertz's solvation entropy correction (**$\Delta G_W^{\ddagger}$** and **$\Delta_r G_W$**) for three representative reactions of different molecularity *m*.

Reaction	$\Delta G^{\ddagger}$	$\Delta G_W^{\ddagger}$	$\Delta_r G$	$\Delta_r G_W$
**2** → **3** via **TS2** (*m* = -1)	15.9	14.8	-11.2	-10.9
**10** → **5** via **TS6** (*m* = 0)	19.5	19.2	-24.2	-24.7
**13** → **5** via **TS9** (*m* = +1)	22.1	22.0	-2.5	-3.1

2. Conformational Search

2.1 Default conformational sampling scheme

Extensive sampling of the conformational space is crucial to properly evaluate a reaction energy profile [1]. "Traditional" manual sampling based on chemical intuition can provide a reasonable starting guess, but the task rapidly becomes unfeasible with the increasing size of the system. Our default approach to sample the conformational space is a locally modified version of the automated search tool in autodE [13] (available in our research group git repository: https://github.com/rahmlab/autodE).This version includes bug fixes and minor improvements.

In a typical autodE run, up to 192 random non-covalent interacting (NCI) complexes (2-5 species) are generated with ETKDGv2, as implemented in RDKit [14]. If the solvate comprises of more than 9 heavy atoms, up to 64 single-molecules conformers are also generated and randomly included in the NCI complexes. All conformers are initially optimized with Gaussian16 at the PCM-B3LYP-D3BJ/4-31G* level of theory, with loose geometry criteria for convergence. Following pruning on geometry and energy criteria, the surviving set is re-optimized with a larger basis set (6-31+G(d,p)). The 5 best structures are finally optimized a third time, alongside a frequency analysis that allows evaluation of thermal corrections. The structure with the lowest DFT (quasi-harmonic-corrected) Gibbs energy is then selected as the best conformer (equation (2), Methods section). In cases where two or more structures are predicted to have similar (within 1.0 kcal/mol) DFT Gibbs energies, the energy refinement was performed on all conformer candidates.

A similar approach for conformational sampling has been applied for TSs. In this case, a set (no less than 4) of preliminary TSs is first obtained by manual search. Then, the automated conformational search is performed on the frozen active site geometry combined with additional HCN molecules (1-5), in the same manner as for NCI complexes. The five lowest energy structure are finally optimized without constraints to a first-order saddle point, and the complex with lowest DFT Gibbs energy is selected.

For each species and TS in our reaction network, we first performed AutodE runs with one and four explicit HCN solvent molecules. To sample intermediate solvation levels (two and three HCNs), we manually add or remove solvent molecules from the complexes found in the AutodE runs, spanning all possible combinations. This approach provides a thorough conformational sampling, while maintaining feasibility. By performing an unbiased sampling with 1 and 4 explicit HCN molecules, we minimize the risk of systematic errors. We occasionally perform full AutodE runs with two, three, or five explicit solvent molecules to ensure exhaustive sampling.

2.2 Enhanced conformational sampling for key reaction steps

For key reaction steps, defined as those with barrier height $\Delta G^{\ddagger} > 24$ kcal/mol, we employ a more rigorous sampling scheme in parallel with AutodE. To this end, we adopt two recently devised algorithms: Global Optimizer Algorithm (GOAT) [15] and SOLVATOR [16], both implemented in ORCA 6.1 [16]. GOAT performs stochastic uphill pushes and downhill optimization on the potential energy surface (PES) in an iterative fashion; SOLVATOR employs the DOCKER algorithm,[16] which is based on a form of Particle Swarm Optimization. Our approach consists of two consecutive workflows:

1. A conformational search on a cluster solvated by a single HCN molecule is first performed using the GOAT algorithm with GFN2-XTB [6] in implicit solvation, by means of the analytical linearized Poisson-Boltzmann (ALPB) model [17] (parameters for water, with a modified dielectric constant $\varepsilon = 144.8$). The best 1-5 structures are subsequently re-optimized at the PCM-B3LYP-D3(BJ)/6-31+G(d,p) level. The structure with the lowest DFT Gibbs energy is selected for the next step. If multiple conformers are within 1 kcal/mol of the minimum, the energy refinement is performed on all candidates.
2. A set of SOLVATOR runs (ALPB-GFN2-XTB) is carried out, adding one additional explicit HCN molecule at each iteration. Similarly to the GOAT workflow, the best 1-5 structures for each solvation level are re-optimized at the PCM-B3LYP-D3(BJ)/6-31+G(d,p) level, and the best candidate (as discussed above) is selected for the next iteration. This cycle is repeated N times to incrementally increase the number of explicit solvent HCN molecules up to N.

Finally, the selected conformers are compared against those from our general AutodE runs. For the final data, we select the single conformer with the lowest DLPNO-CCSD(T)-refined Gibbs energy from all methods.

2.3 Validating modeling of polyimine-facilitated redox reaction step

In our polyimine-facilitated step reaction (**TS17**), we use a pentamer fragment to model the polymer. We limit our model both because shorter polyimine oligomers are more likely to persist than long polymers, and because sufficient conformational sampling of even larger models is practical unfeasible. To test the stability of our results we have computed the same reaction step using a polyimine tetramer and hexamer, each solvated by a single explicit HCN solvent molecule. The resulting predicted barrier heights shown in Table S4, and they do not vary appreciably with the size of polyimine fragment.

Table S4: Barrier height ($\Delta G^{\ddagger}$) of the polyimine-facilitated step reaction (**TS17**), calculated with different sizes of the polyimine fragment. These values are computed using a single explicit HCN solvent molecule. In our full reaction model, we use the polyimine pentamer, and a cluster solvated by four explicit HCN molecules (hence the different barrier, see Figure S7).

**Polyimine size**	**$\Delta G^{\ddagger}$, TS17**
Tetramer	26.7
Pentamer	26.7
Hexamer	26.3

3. Validation of DLPNO-CCSD(T)

In our work, we use the domain-based local pair natural orbital coupled-cluster method with perturbative triples (DLPNO-CCDS(T)) [18] to refine electronic energies. DLPNO-CCSD(T) is a near-linear scaling approximation to full CCSD(T), the gold standard of quantum chemistry, that retains 99% of the CCSD(T) correlation energy

[18]. Several benchmarks have reported “DLPNO errors” that are within 1 kcal/mol [19], [20], [21]. To assess this methods reliability for our systems, we carried out a comparison between DLPNO-CCSD(T) and CCSD(T) on the initiation of the HCN oligomerization: the cyanide nucleophilic attack on HCN to give IAN (**2**), through **TS1**, and the cyanide nucleophilic attack on **2** to give AMN (**3**), through **TS2,** each cluster solvated by a single explicit HCN solvent molecule. The two methods provide very similar Gibbs energy profiles, as shown in Figure S9. Figure S9 also demonstrates that DFT of the B3LYP-flavor clearly underestimates both reaction barriers while overestimating the spontaneity of the first reaction.

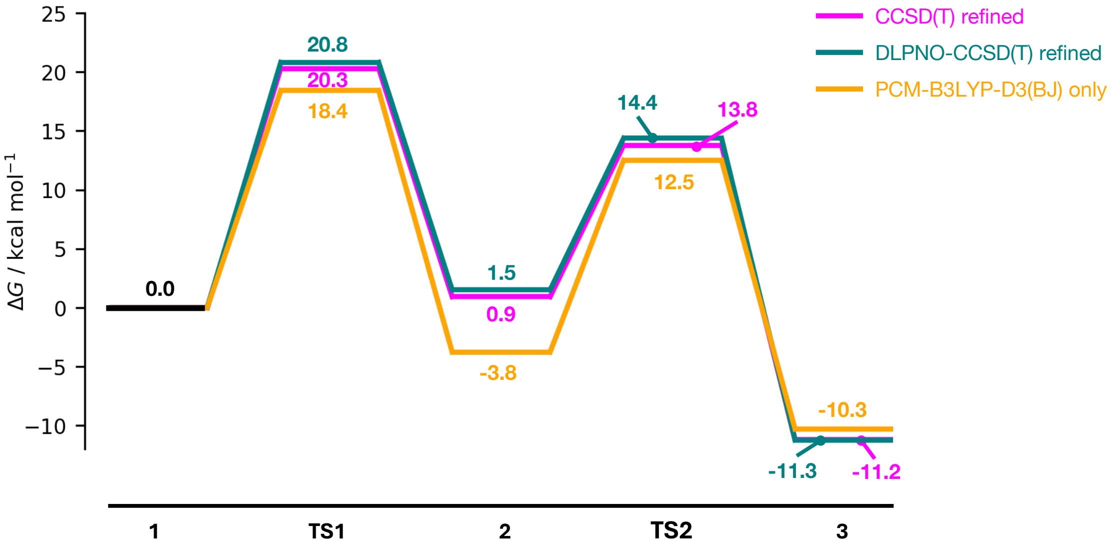

Figure S9: Gibbs energy profile of AMN formation from HCN (**1** → **3** via **TS1**, **2**, and **TS2**), modeled with a single explicit HCN solvent molecule. Geometries and thermal corrections to the electronic energy have been calculated at the PCM-B3LYP-D3BJ/6-31+G(d,p) level of theory. Electronic energies and HCN solvation energy ($\Delta G^*_{solv}(HCN)$) have been obtained with B3LYP-D3BJ/6-31+G(d,p) (orange), DLPNO-CCSD(T)/aug-cc-pVTZ (teal) and CCSD(T)/aug-cc-pVTZ (magenta).

4. Analysis of Contributions to Relative Gibbs Energy Estimates

Here we analyze how relative Gibbs energies can be affected by the many individual corrections to electronic structure, vibrational energy terms, and solvation that are part of our composite approach. For this analysis we consider two reactions: the HCN addition to DAMN (**4** → **12** via **TS10**), and the six-member ring formation from **11** to adenine (**11** → **6** via **TS8)**. It should be noted that the magnitude of these corrections necessarily varies for other reactions.

Tables S5-S6 report the reaction Gibbs energies and barrier heights (in kcal/mol), showing the progression from the PCM-B3LYP/6-31+G(d,p) level of theory with one explicit HCN solvent molecule to our best estimates. The data indicates that explicit solvation beyond a single HCN and DLPNO-CCSD(T) refinement of the electronic energy are the most impactful. However, these corrections are not systematic: their size and sign depend on the specific reaction step.

For the HCN addition (**4** → **12** via **TS10**), explicit solvation has only a minor effect on the barrier height, whereas DLPNO-CCSD(T) substantially changes both the barrier and the reaction free energy (Table S5). In contrast, for the cyclization step (**11** → **6** via TS8, Table S6), convergence with respect to explicit solvation greatly stabilizes the transition state. These examples illustrate that reliable relative Gibbs energies in this system require explicit consideration of both high-level electronic structure refinement and carefully convergence of energies with respect to the number of explicit solvent molecules.

Table S5: Impact of different Gibbs energy refinement steps for the reaction **4** → **12** via **TS10**.

**Refinements for 4 → 12 via TS10**	**ΔG‡**	**ΔΔG‡**	**Δ_rG**	**ΔΔ$_r$G‡**
PCM-B3LYP-D3(BJ)/6-31+G(d,p)	**28.4**	-	**-11.1**	-
RHHO correction	**27.8**	*-0.6*	**-11.2**	*-0.1*
DLPNO-CCSD(T) refinement	**26.1**	*-1.7*	**-8.2**	*+3.0*
DLPNO-CCSD(T) refinement, including solvent-induced relaxation, ΔE_{relax}	**26.0**	*-0.1*	**-8.1**	*+0.1*
Convergence with respect to explicit solvation	**25.8**	*-0.2*	**-4.4**	*+3.7*
**Total**		***-2.6***		***+6.7***

Table S6: Impact of each refinement step on the relative Gibbs energies for the reaction **11** → **6** via **TS8**.

**Refinements for 11 → 6 via TS8**	**ΔG‡**	**ΔΔG‡**	**Δ_rG**	**ΔΔ$_r$G‡**
PCM-B3LYP-D3(BJ)/6-31+G(d,p)	**25.3**	-	**-18.5**	-
RHHO correction	**25.3**	*0.0*	**-19.0**	*-0.5*
DLPNO-CCSD(T) refinement	**27.5**	*+2.2*	**-16.9**	*+2.1*
DLPNO-CCSD(T) refinement, including solvent-induced relaxation, ΔE_{relax}	**27.9**	*+0.4*	**-16.3**	*+0.6*
Convergence with respect to explicit solvation	**20.9**	*-7.0*	**-18.9**	*-2.6*
**Total**		***-4.4***		***-0.4***

5. Sensitivity to Cyanide Concentration

Concentration effects on the barrier heights are accounted for via the standard concentration correction (Eq. 4), such that at 278 K increasing $[CN^-]$ from 1.0 to 1.1 M gives $RTln(1.1) \approx$ +0.053 kcal/mol, while decreasing it to 0.9 M gives $RTln(0.9) \approx$ -0.058 kcal/mol. These shifts are negligible compared with the ±1 kcal/mol uncorrelated barrier uncertainty used for sensitivity estimates. A barrier shift comparable to ±1 kcal/mol at 278 K would require $[CN^-]$ to vary to roughly 0.2–6 M.

6. Proton Transfer and Tautomerization

Several reaction pathways studied in this work involve proton transfer (PT) steps mediated by solvent HCN molecules, which can either proceed in a stepwise or concerted manner. In case of a stepwise mechanism, the barrier heights we compute for PT steps never exceed 10 kcal/mol, and are always smaller than that of other steps, such as nucleophilic attacks or ring-forming reactions. PT reaction steps can thus be assumed to be rapid and are omitted in the reaction profiles.

Figure S10 shows the DFT reaction energy profile obtained from two intrinsic reaction coordinate (IRC) calculations for a representative stepwise nucleophilic attack: the addition of HCN to DAMN (**4**). This process is initiated by a PT that generates a negatively charged nitrogen, which subsequently attacks the nitrile group of HCN (**TS10**). We find that this anionic pathway results in a lower reaction path maximum compared to an attack by a

neutral amino group. The PT step is much faster than the nucleophilic attack, thus it does not contribute to the kinetics of the process. An IRC calculation of the same reaction solvated by single HCN molecule (gray circles) is shown for comparison. The addition of additional explicit solvent molecules does not alter the path qualitatively.

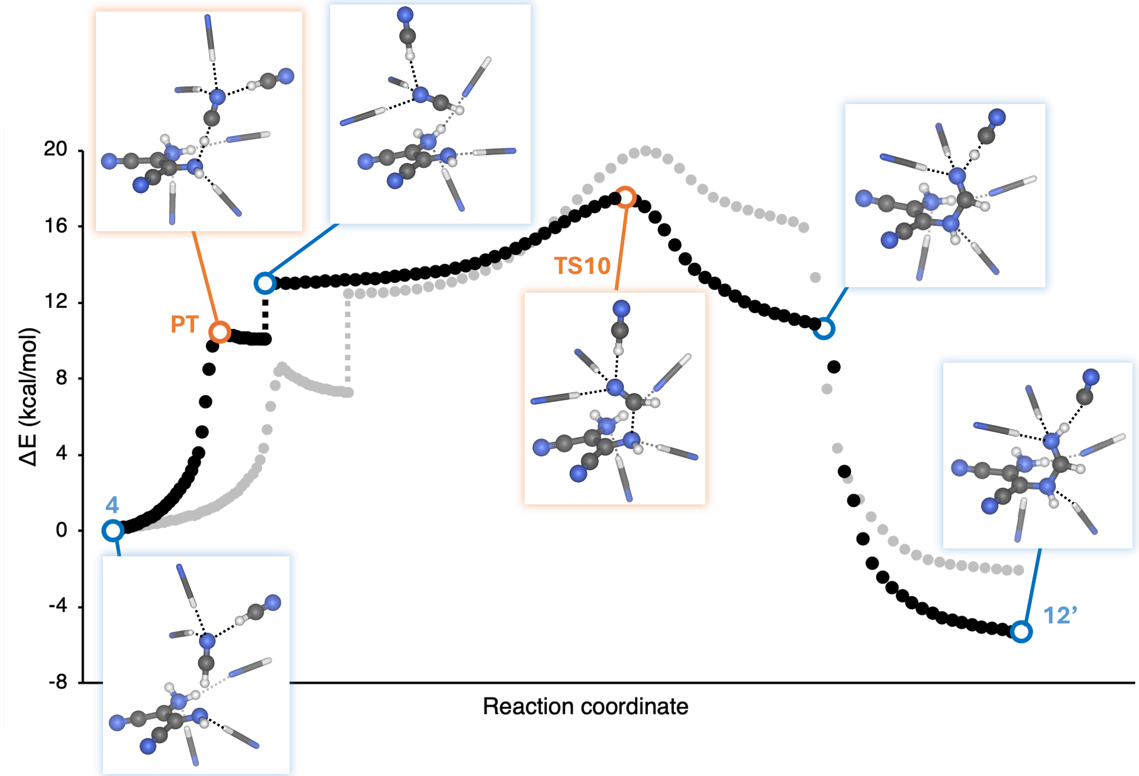

Figure S10: Intrinsic reaction coordinate (IRC) path (DFT energy in kcal/mol, black circles) from **4** to **12’** via proton transfer (PT) and **TS10**. Explicit solvent molecules are represented as sticks. The IRC path of the same reaction but with a single explicit HCN solvent molecule (gray circles) is shown for comparison. TSs are colored in orange. The vertical leaps in energy (dashed lines) is due to a conformational change.

Imine-enamine tautomerization occur frequently in our explored reaction network. These processes involve two PT steps, and a positively charged intermediate (Figure S11). Given the fast kinetics of PT steps, we assume that imine-enamine equilibria are always maintained. The enamine tautomer is always predicted to be favored, and the energy difference is such that virtually no imine form is present in the reaction medium. For example, the Gibbs energy difference is 4.5 kcal/mol for the **10’⇌10** equilibrium, and 9.8 kcal/mol for **6’⇌6** (Figures S13-S15**)**. For ease of interpretability, we have therefore omitted the imine form in our reaction profiles (Figures 1-5) in the main text.

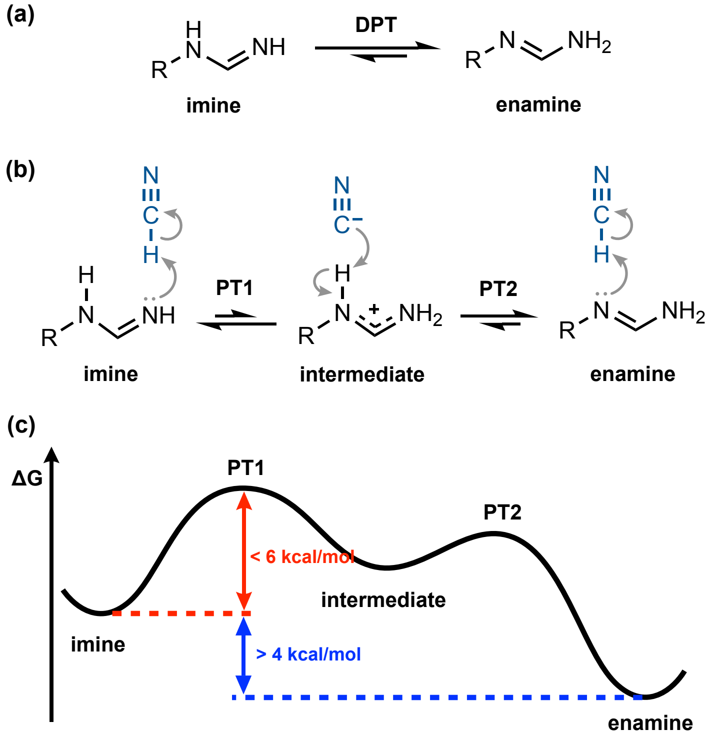

Figure S11: Imine-enamine tautomerization of an amidine group, as encountered in our work. (a) Overall reaction, involving a double proton transfer (DPT), also indicating the favored enamine form at equilibrium. (b) detailed mechanism of the tautomerization mediated by a solvent HCN molecule. (c) Example of a Gibbs energy profile of the process.

Figure S12 illustrates the DFT reaction energy profile from AMN (**3**) to product **10**, determined by two IRC calculations, including a single explicit HCN solvent molecule, for simplicity. The reaction begins with a nucleophilic attack of AMN on HCN (**TS5**) to form the intermediate **10'**. This step involves a concerted double proton transfer mediated by HCN and CN^-. Intermediate **10'** then tautomerizes to product **10** via a stepwise, double proton transfer mechanism (**PT1** and **PT2**). This latter step is considerably faster than the initial nucleophilic attack.

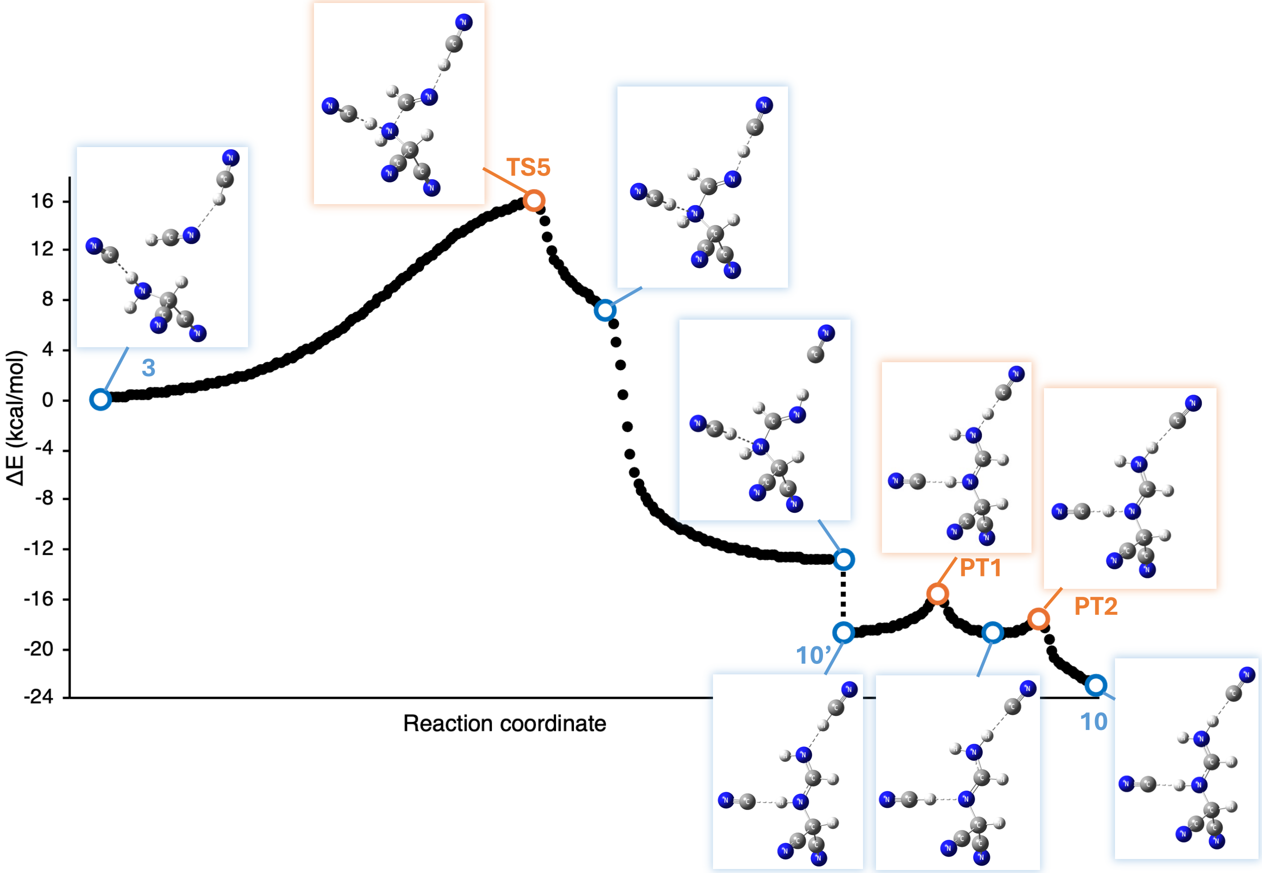

Figure S12: Intrinsic reaction coordinate (IRC) paths (DFT energy in kcal/mol) from **3** to **10'** via **TS5**, and from **10'** to **10** via tautomerization (**PT1** and **PT2**). TSs are colored in orange. The vertical leap in energy for **10'** (dashed line) is due to a conformational change.

Gibbs energy reaction profiles including tautomerization processes are presented in Figures S13-S15. There, only the highest PT-barrier out of the two are shown and the charged intermediate is omitted. Note that those TS are labeled as DPT - for double proton transfer - followed by the number of the TS preceding them. Note also that all relative energies in Figures S13-S15 differ slightly from those in Figures 1-5 of the main text. This stems from the impossibility of optimizing most PT TSs in vacuum, which necessitates us to omit consideration of solvent-induced relaxation effects term, ΔE_{relax} in all our calculations of solvation energy for PT steps (see the Methods section, eq. (3)). The relative Gibbs energies in Figures S13-S15 are therefore likely to be less accurate compared to those in Figures 1-5 of the main text.

Some of the Gibbs reaction barriers connecting the charged intermediate and the enamine form (**PT2** in Figure S10) are calculated to have a slight negative height (> -1.0 kcal/mol). This underestimation is an artifact arising when dealing with very small barriers (heights < 1-2 kcal/mol), for which the ideal-gas approximation, the missing vibrational mode in the partition function, and the lack of the ΔE_{relax} term (described in the Eq. 3 of the method section in the main text) may result in an error in the Gibbs energy that is greater than the barrier itself. We emphasize that the overall kinetics of the pathways to adenine are not sensitive to these reaction steps.

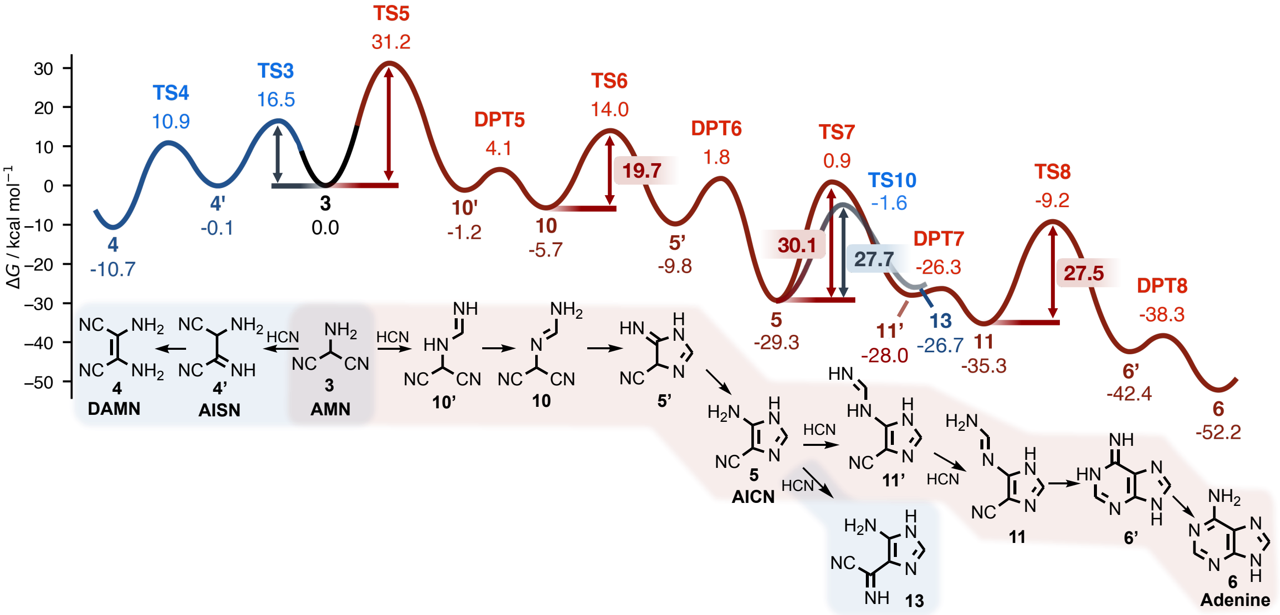

Figure S13: Gibbs energy profile including tautomerization (double proton transfer, DPT) processes of the AMN pathway shown in red (cfr. Figure 2 in the main text). DAMN formation is also shown for a comparison of kinetics. Energies in kcal/mol are provided relative to AMN, except for calculations of selected barrier heights that are shown inside boxes. All energies are here calculated including a single solvent HCN molecule and without consideration of the ΔE_{relax} term, described in Eq. (3).

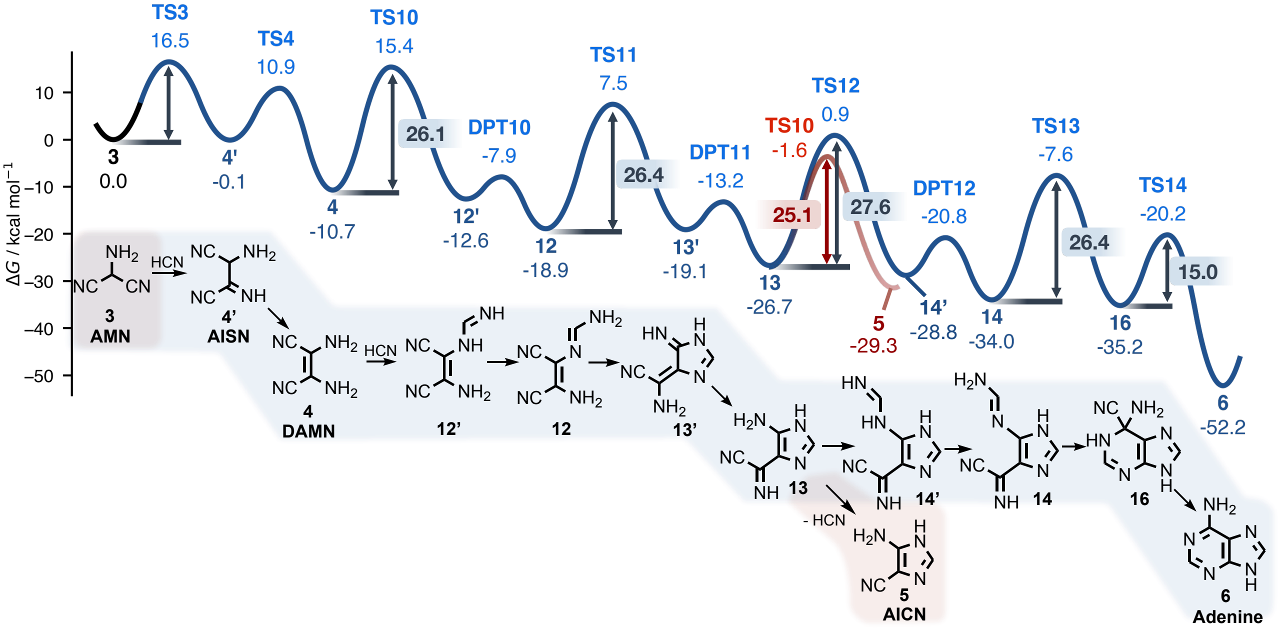

Figure S14: Gibbs energy profile including tautomerization (double proton transfer, DPT) processes of the DAMN pathway shown in blue (cfr. Figure 3 in the main text). Energies in kcal/mol are provided relative to AMN, except for calculations of selected barrier heights that are shown inside boxes. All energies are here calculated including a single solvent HCN molecule and without consideration of the ΔE_{relax} term, described in Eq. (3).

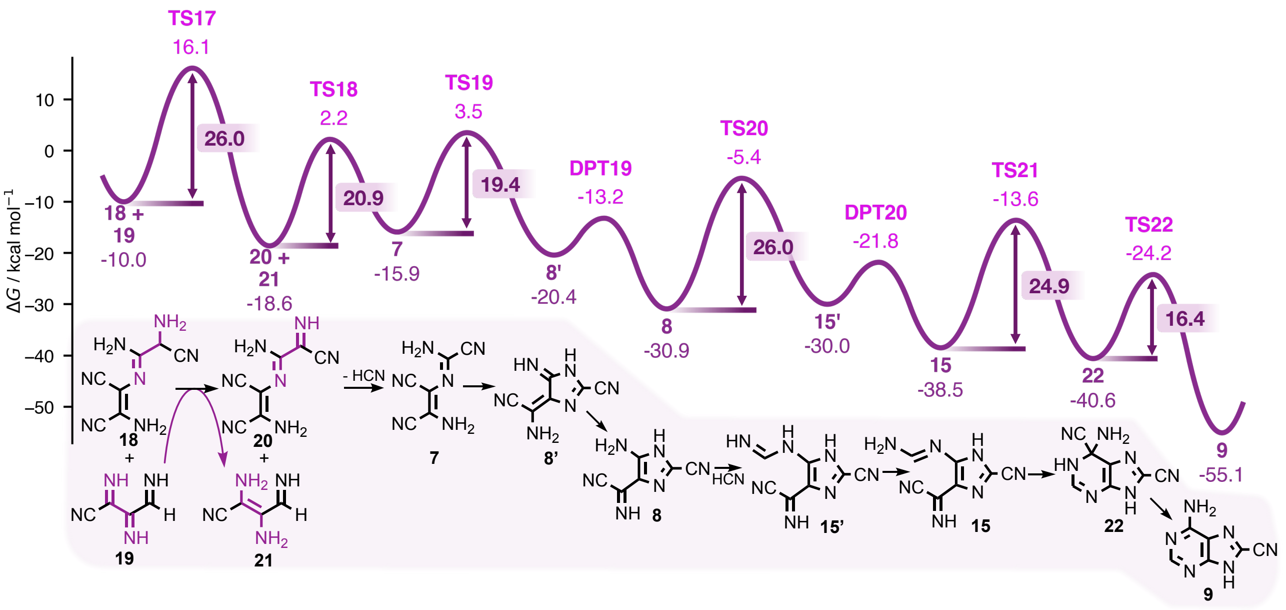

Figure S15: Gibbs energy profile including tautomerization (double proton transfer, DPT) processes of the Voet and Schwartz's (VS) pathway, from intermediate **18** (cf. Figure 5 in the main text). Energies in kcal/mol are provided relative to AMN, except for calculations of selected barrier heights that are shown inside boxes. All energies are here calculated including a single solvent HCN molecule and without consideration of the ΔE_{relax} term, described in Eq. (3).

7. Microkinetic Modeling

7.1 Kinetics Equations

In our microkinetic modeling the reaction network is represented by a system of differential equations, one for each reaction step, as provided in Table S7. These kinetics equations have been numerically solved using the Livermore Solver for Ordinary Differential equations with Automatic method switching for stiff and non-stiff problems (LSODA) [22] and the Backward Differentiation Formula (BDF) method [23], as implemented in SciPy version 1.13.1. A script describing these calculations are provided in the SND repository linked in the beginning of this document. Reaction rate constants have been calculated using the Eyring equation. For a reaction R of the form:

$$A \rightleftharpoons B \tag{S5}$$

the forward reaction rate constant, $k_{f,R}$, is given by:

$$k_{f,R} = \frac{k_B T}{h} \exp\left(\frac{\Delta G_R^\ddagger}{RT}\right) \quad , \tag{S6}$$

where k_B is the Boltzmann constant, T is the temperature (278 K), h the Planck's constant, R the gas constant, and $\Delta G_R^\ddagger$ the Gibbs energy barrier. The backward reaction rate constant, $k_{b,R}$, is given by:

$$k_{b,R} = \frac{k_B T}{h} \exp\left(\frac{\Delta G_R^\ddagger - \Delta_r G_R}{RT}\right) \tag{S7}$$

Where $\Delta_r G_R$ is the reaction Gibbs energy for the reaction. The reaction rate $r_R(t)$ is then:

$$r_R(t) = k_{f,R}[A]_t - k_{b,R}[B]_t, \tag{S8}$$

where $[A]_t$ and $[B]_t$ are the concentrations of species A and B, respectively, at time t. The parameters used for our reaction network are shown in Table S7. Gibbs energies are corrected to a standard state concentration of 1 M for

all species, including HCN, which is why the values in Table S7 differ somewhat from Figures 1-5 and the values reported in the main text. The initial conditions for t = 0 have been set to $[HCN]_0 = 26.05\ M$ and 0 M for every other species in the reaction network.

Table S7: Reaction species and parameters (in kcal/mol) of our reaction network. Nomenclature and numbering as in the main text, except for "p-Imine" (polyimine) and "red-p-Imine" (reduced polyimine).

R	TS	Reac 1	Reac 2	Prod 1	Prod 2	ΔG‡(278K)	Δ_rG(278K)
1	**TS1**	HCN	HCN	IAN		25.808	3.971
2	**TS2**	IAN	HCN	AMN		20.176	-8.566
3	**TS5**	AMN	HCN	**10**		31.282	-3.024
4	**TS6**	**10**		AICN		16.798	-25.396
5	**TS7**	AICN	HCN	**11**		30.144	-3.309
6	**TS8**	**11**		Adenine		20.860	-18.913
7	**TS3**	AMN	HCN	AISN		17.047	0.3850
8	**TS4**	AISN		DAMN		11.212	-10.611
9	**TS10**	DAMN	HCN	**12**		27.600	-2.613
10	**TS11**	**12**		**13**		20.822	-12.911
11	**TS9**	**13**		AICN	HCN	23.893	-2.669
12	**TS12**	**13**	HCN	**14**		28.080	-3.767
13	**TS13**	**14**		**16**		21.585	-3.193
14	**TS14**	**16**		Adenine	HCN	14.131	-17.930
15	**-**	HCN	HCN	p-Imine		22.226	2.755
16	**TS15**	DAMN	AISN	**17**		17.318	-5.162
17	**TS16**	**17**		**18**	HCN	13.532	-6.763
18	**TS17**	**19**	p-Imine	**20**	red-p-Imine	25.304	-3.401
19	**TS18**	**21**		**7**	HCN	20.857	1.140
20	**TS19**	**7**		**8**		17.402	-14.724
21	**TS20**	**8**	HCN	**15**		25.606	-6.444
22	**TS21**	**15**		**22**		21.304	-8.429
23	**TS22**	**22**		**9**	HCN	21.544	-15.048

7.2 Monte Carlo Sampling of Reaction Parameters

For our Monte Carlo analysis of the reaction kinetics, we have run ~50000 simulations, where each parameter in Table S7 was independently sampled from a normal distribution centered at the calculated value, with standard deviation σ = *1.0* kcal/mol. In order words, for a kinetics simulation i, the barrier height $\Delta G_i^{\ddagger}(R)$, and reaction energy $\Delta_{\mathrm{r}} G_i(R)$ for each reaction R are obtained by:

$$\Delta G_i^{\ddagger}(R) = \Delta G^{\ddagger}(R) + \{X \sim \mathcal{N}(\mu = 0, \sigma^2 = 1)\} \quad \text{(S9)}$$

$$\Delta_{\mathrm{r}} G_i(R) = \Delta_{\mathrm{r}} G(R) + \{X \sim \mathcal{N}(\mu = 0, \sigma^2 = 1)\} \quad \text{(S10)}$$

Where $\mathcal{N}(\mu = 0, \sigma^2 = 1)$ denotes a normal distribution with mean $\mu=0$ and variance $\sigma^2=1$ (and thus standard deviation $\sigma=1$), in kcal/mol, and $X \sim \mathcal{N}$ represents a random value sampled from $\mathcal{N}$. Figure S16 shows adenine concentration distribution at $t = 7.92 \cdot 10^8$ s ≈ 25 years. The 16th percentile (15.9%) is $6 \cdot 10^{-5}$ M, whereas the 84th percentile (84.1%) is $5 \cdot 10^{-2}$ M. Results for adenine and product **9** after 25 years are compared in Figure S17, which

shows that the broader band for **9** is given by its larger negative skewness. Finally, the evolution of adenine concentration distribution over time is plotted in Figure S18.

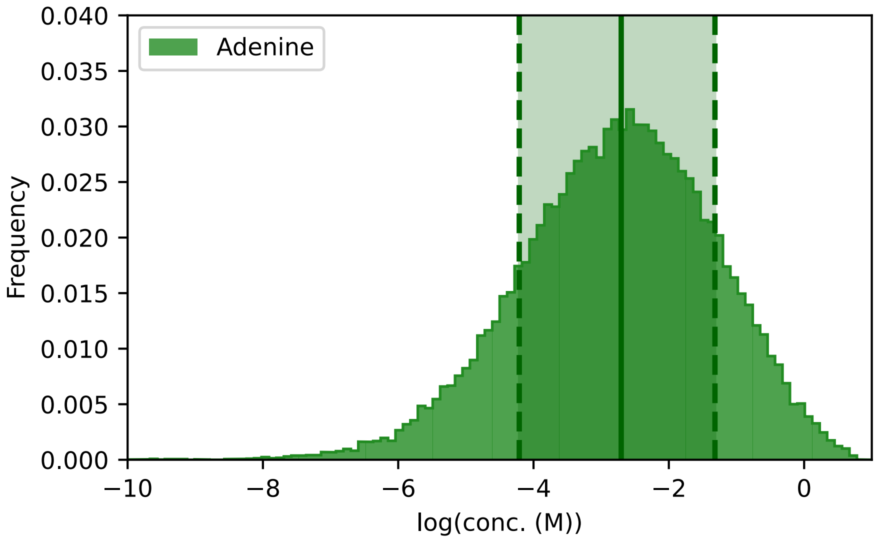

Figure S16: Predicted distribution of formed adenine (log_{10} molar concentration) from ~50000 Monte Carlo runs after $7.32 \cdot 10^8$ s ≈ 25 years. The distribution consists of 100 bins, ranging from 10^{-10} M to 1 M. The colored area within the vertical dashed lines represents 68.3% of the simulations, while the vertical solid line is the median.

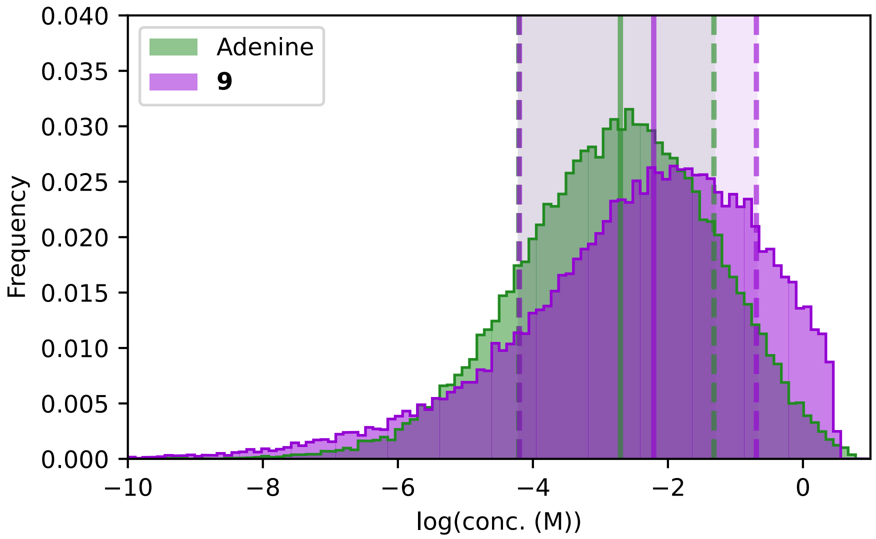

Figure S17: Predicted distribution of formed adenine, in green, and **9**, in purple, (log_{10} molar concentration) from ~50000 Monte Carlo runs after $7.32 \cdot 10^8$ s ≈ 25 years. Both distributions consist of 100 bins, ranging from 10^{-10} M to 1 M. The colored areas (green for adenine, purple for **9**) within the vertical dashed lines represents 68.3% of the simulations, while the vertical solid lines are the medians.

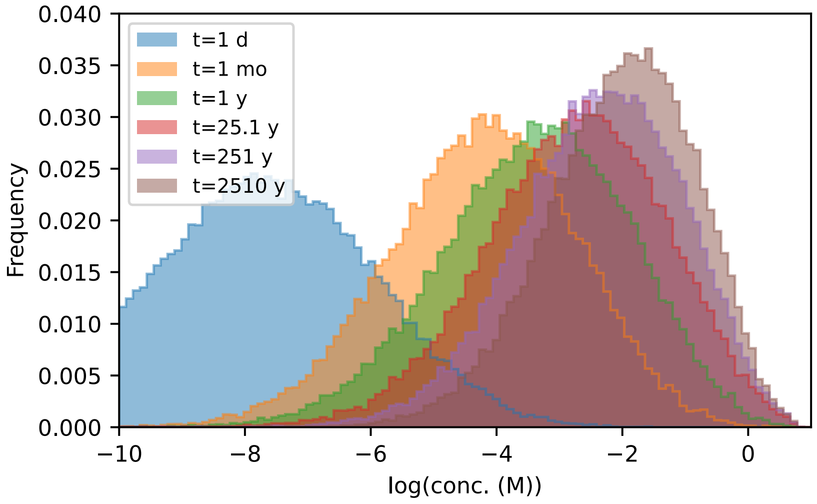

Figure S18: Predicted distributions of formed adenine ($\log_{10}$ molar concentration) from the Monte Carlo runs at different times t, expressed in days (d), months (mo), or years (y). Each distribution consists of 100 bins, ranging from 10^{-10} M to 1 M.

7.3 Contributions to the Rate of Formation of AICN and Adenine

Multiple reaction pathways contribute to both AICN and adenine formation. The fractional contributions of these different pathways (Figure 6b-c) are obtained from the forward reaction rates. In general, for a species X formed by N different reactions, the fractional contribution of the species A to X formation at time t, $f_{A\to X}(t)$, is calculated as:

$$f_{A\to X}(t) = \frac{r_{A\to X}(t)}{\sum_i^N r_{i\to X}(t)} \quad \text{(S11)}$$

where $r_{A\to X}(t)$ is the forward rate of the reaction $A \to X$ at time t. From the Monte Carlo sampling, we obtain a distribution of $r_{A\to X}(t)$ (Figure S19) and $f_{A\to X}(t)$ (X=AICN, adenine). In the case of adenine, the distribution of fractional contributions is quite broad, hence our choice to narrow down the highlighted bands (Figure 6c) to 38.3% ($\sigma = 0.5$) of distributions around the median, instead of 68.3%, to ease readability. Figure S19 illustrates an example at t ≈ 25 years, for which the revised DAMN pathway contributes little to adenine formation, while the VS path is predicted to contribute the most.

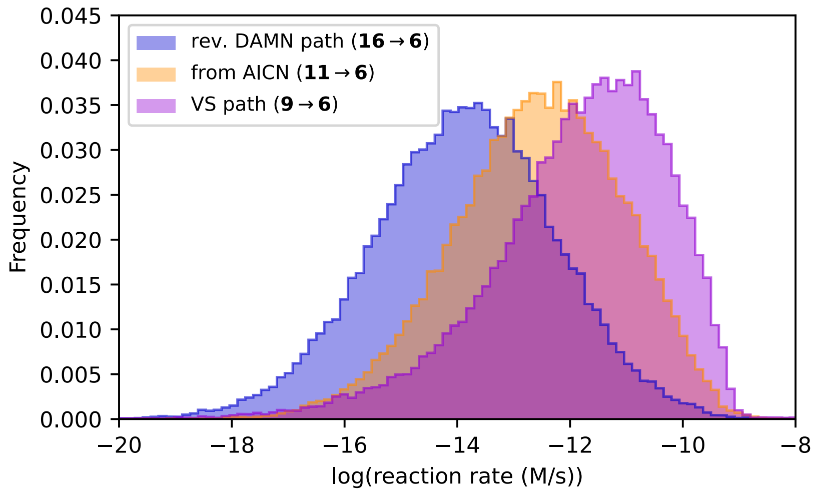

Figure S19: Predicted distribution of adenine formation rates ($\log_{10}$ molar per second, M/s) from **16** (blue), **11** (orange), and **9** (purple), from the Monte Carlo runs at t ≈ 25 years.

7.4 Polymerization of Polyimine

We have assumed that polyimine is formed through a chain-growth (CG) mechanism, with a forward rate that is proportional to the square of the concentration of the monomer (HCN):

$$r_{f,CG} = k_{f,CG}[HCN]^2 \quad \text{(S12)}$$

the forward rate constant $k_{f,CG}$ is obtained from the barrier of the initiation step, the nucleophilic addition of CN^- to the nitrile group of IAN (**2**). The reverse rate constant is given by such barrier, minus the Gibbs energy change associated with the addition of one monomer to the polymer (equation S7), $\Delta G_{HCN}(polyimine)$, which we obtain through the formula:

$$\Delta G_{HCN}(polyimine) = \frac{G_{n+m} - G_n}{m} - G(HCN), \quad \text{(S13)}$$

Where G_{n+m} and G_n are the Gibbs energy of polyimine composed of $n+m$ and n HCN units, respectively. For this estimate, we used n=19 and m=2, and $\Delta G_{HCN}(polyimine)$ = +0.95 kcal/mol.

7.5 Adenine yield against experiments

Our predicted yields of adenine provided in Table S8 shows excellent agreement with experimental yields from aqueous HCN polymerization experiments, shown in Table S9. Table S9 shows selected examples from the literature including the reported reaction conditions, including the hydrolysis step on the HCN-derived products, when carried out. This comparison should be interpreted with caution, as the experimental conditions vary widely across studies, and are different from our model system of pure HCN. Adenine yield strongly depends on temperature, initial reagents, hydrolysis conditions, as well as the analytical techniques employed to identify it.

Table S8: Results of our Monte Carlo sampling of the microkinetic modeling at 278 K. Shown is the median of the yield of adenine (**6**), as a mol percentage of initial HCN, when no hydrolysis is carried out (central column, VS pathway halts at **9**), and assuming hydrolysis conditions that quantitatively convert product **9** to **6** (right column).

**Time**	**Median adenine yield (mol %), no hydrolysis**	**Median adenine yield (mol %), assuming fast hydrolysis of 9**
1 day	$3 \cdot 10^{-7}$ %	$3 \cdot 10^{-6}$ %
1 week	0.00009%	0.00009%
1 month	0.001%	0.001%
1 year	0.01%	0.02%
25 years	0.04%	0.2%
30 ky	0.32%	1.3%

Table S9: Reported adenine yield from various selected experiments, including the experimental conditions.

Reagents	Solvent	Temp.	Time	Hydrolysis step	Adenine yield (mol %)	Ref.
HCN + TEA	Methanol	RT	3 d	None	Detected	[24]
HCN + trace NH_3	HCN	RT	3 d	None	Detected	[24]
Gaseous HCN on MgO surfaces	-	-123°C to 27°C	-	None	Detected	[25]
HCN	Ammonia	120°C	20 h	None	15%	[26]
HCN	Ammonia	80°C	20 h	None	22%	[27]
Sat. HCN + NH_4OH	Water	90°C	24 h	Acid, 6 M HCl overnight	Detected	[28]
NH_4CN 10 M	Water	80°C	24 h	Acid, 6 M HCl 24h	0.027%	[29]
NaCN 0.1 M	Water	-30°C	2 mo	Acid, 6 M HCl 24h	0.0004%	[29]
NH_4CN 0.1 M	Water	-20°C	25 y	Acid, 6 M HCl 24h	0.038%	[29]
NH_4CN 0.1 M	Water	RT	1 w	Acid, 6 M HCl 100°C 24h	0.000013%	[30]
NH_4CN 0.1 M	Water	RT	4 w	Acid, 6 M HCl 100°C 24h	0.00031%	[30]
NH_4CN 0.1 M	Water	RT	8 w	Acid, 6 M HCl 100°C 24h	0.00062%	[30]
NH_4CN 0.001 M	Water	-20°C	3 mo	Acid, 6 M HCl 100°C 24h	0.0042%	[30]
NH_4CN 0.01 M	Water	-20°C	3 mo	Acid, 6 M HCl 100°C 24h	0.01%	[30]
NH_4CN 0.1 M	Water	-20°C	3 mo	Acid, 6 M HCl 100°C 24h	0.0094%	[30]
NH_4CN 0.1 M	Water	-78°C	27 y	pH 8, 0.01 M Na_3PO_4 140°C 3 d	0.012%	[30]
NH_4CN 0.1 M	Water	-78°C	27 y	Acid, 6 M HCl 100°C 24h	0.029%	[30]
NH_4CN 0.1 M	Water	-78°C	27 y	None	0.00016%	[30]
HCN 0.1 M + NH_4OH to pH 9.2	Water	RT	4-12 mo	Acid, 6 M HCl 110°C 24h	0.003-0.004%	[31]
NH_4CN 10 M	Water	80°C	2 d	Acid, 6 M HCl 100°C 24h	0.028%	[32]
NH_4CN 0.1 M	Water	-20°C	2 mo	Acid, 6 M HCl 100°C 24h	0.005%	[32]
NH_4CN 0.1 M	Water	-20°C	25 y	Acid, 6 M HCl 100°C 24h	0.035%	[32]
NH_4CN 0.1 M	Water	-78°C	25 y	Acid, 6 M HCl 100°C 24h	0.040%	[32]
HCN 0.01 M + NH_4OH to pH9.2 + glyconitrile	Water	-2°C	98 d	Acid 5 M HCl 100°C 18 h	0.004%	[33]

8. References

[1] H. Ryu, J. Park, H. K. Kim, J. Y. Park, S. T. Kim, and M. H. Baik, “Pitfalls in Computational Modeling of Chemical Reactions and How to Avoid Them,” *Organometallics*, vol. 37, no. 19, pp. 3228–3239, Oct. 2018, doi: 10.1021/ACS.ORGANOMET.8B00456.

[2] V. S. Bryantsev, M. S. Diallo, and W. A. Goddard, “Calculation of solvation free energies of charged solutes using mixed cluster/continuum models,” *Journal of Physical Chemistry B*, vol. 112, no. 32, pp. 9709–9719, Aug. 2008, doi: 10.1021/JP802665D.

[3] M. Sánchez, P. F. Provasi, G. A. Aucar, I. Alkorta, and J. Elguero, “Theoretical study of HCN and HNC neutral and charged clusters,” *Journal of Physical Chemistry B*, vol. 109, no. 38, pp. 18189–18194, Sep. 2005, doi: 10.1021/jp052935d.

[4] D. P. Freitas, F. N. N. Pansini, and A. J. C. Varandas, “Linear and cyclic (HCN)n clusters: A DFT study of IR and Raman spectra,” *Chem Phys Lett*, vol. 828, p. 140734, Oct. 2023, doi: 10.1016/j.cplett.2023.140734.

[5] P. Pracht, F. Bohle, and S. Grimme, “Automated exploration of the low-energy chemical space with fast quantum chemical methods,” *Physical Chemistry Chemical Physics*, vol. 22, no. 14, pp. 7169–7192, Apr. 2020, doi: 10.1039/C9CP06869D.

[6] C. Bannwarth, S. Ehlert, and S. Grimme, “GFN2-xTB - An Accurate and Broadly Parametrized Self-Consistent Tight-Binding Quantum Chemical Method with Multipole Electrostatics and Density-Dependent Dispersion Contributions,” *J Chem Theory Comput*, vol. 15, no. 3, pp. 1652–1671, Mar. 2019, doi: 10.1021/ACS.JCTC.8B01176.

[7] M.J. Frisch and et al., “Gaussian 16, Revision B.01,” 2016, *Wallingford CT*.

[8] L. Börnstein, *Vapor Pressure and Antoine Constants for Nitrogen Containing Organic Compounds*, vol. 20C. Springer-Verlag, 2001. doi: 10.1007/B88812.

[9] J. N. Harvey, F. Himo, F. Maseras, and L. Perrin, “Scope and Challenge of Computational Methods for Studying Mechanism and Reactivity in Homogeneous Catalysis,” *ACS Catal*, vol. 9, no. 8, pp. 6803–6813, Aug. 2019, doi: 10.1021/ACSCATAL.9B01537.

[10] D. H. Wertz, “Relationship between the Gas-Phase Entropies of Molecules and Their Entropies of Solvation in Water and 1-Octanol,” *J Am Chem Soc*, vol. 102, no. 16, pp. 5316–5322, 1980, doi: 10.1021/JA00536A033.

[11] M. W. Chase, *NIST-JANAF Thermochemical Tables*, 4th ed. American Institute of Physics, 1998.

[12] W. F. Giauque and R. A. Ruehrwein, “The Entropy of Hydrogen Cyanide. Heat Capacity, Heat of Vaporization and Vapor Pressure. Hydrogen Bond Polymerization of the Gas in Chains of Indefinite Length,” *J Am Chem Soc*, vol. 61, no. 10, pp. 2626–2633, Oct. 1939, doi: 10.1021/ja01265a017.

[13] T. A. Young, J. J. Silcock, A. J. Sterling, and F. Duarte, “autodE: Automated Calculation of Reaction Energy Profiles— Application to Organic and Organometallic Reactions,” *Angewandte Chemie International Edition*, vol. 60, no. 8, pp. 4266–4274, Feb. 2021, doi: 10.1002/ANIE.202011941.

[14] G. Landrum and et al., “RDKit 2023.03.2,” 2023.

[15] B. de Souza, “GOAT: A Global Optimization Algorithm for Molecules and Atomic Clusters,” *Angewandte Chemie International Edition*, vol. 64, no. 18, p. e202500393, Apr. 2025, doi: 10.1002/ANIE.202500393.

[16] F. Neese, “Software Update: The ORCA Program System—Version 6.0,” *Wiley Interdiscip Rev Comput Mol Sci*, vol. 15, no. 2, p. e70019, Mar. 2025, doi: 10.1002/WCMS.70019.

[17] S. Ehlert, M. Stahn, S. Spicher, and S. Grimme, "Robust and efficient implicit solvation model for fast semiempirical methods," *J Chem Theory Comput*, vol. 17, no. 7, pp. 4250–4261, Jul. 2021, doi: 10.1021/ACS.JCTC.1C00471.

[18] C. Riplinger and F. Neese, "An efficient and near linear scaling pair natural orbital based local coupled cluster method," *J Chem Phys*, vol. 138, no. 3, Jan. 2013, doi: 10.1063/1.4773581.

[19] M. Gray and J. M. Herbert, "Assessing the domain-based local pair natural orbital (DLPNO) approximation for non-covalent interactions in sizable supramolecular complexes," *Journal of Chemical Physics*, vol. 161, no. 5, p. 54114, Aug. 2024, doi: 10.1063/5.0206533/3306675.

[20] I. Sandler, J. Chen, M. Taylor, S. Sharma, and J. Ho, "Accuracy of DLPNO-CCSD(T): Effect of basis set and system size," *Journal of Physical Chemistry A*, vol. 125, no. 7, pp. 1553–1563, Feb. 2021, doi: 10.1021/ACS.JPCA.0C11270.

[21] S. Mallick, B. Roy, and P. Kumar, "A comparison of DLPNO-CCSD(T) and CCSD(T) method for the determination of the energetics of hydrogen atom transfer reactions," *Comput Theor Chem*, vol. 1187, p. 112934, Oct. 2020, doi: 10.1016/J.COMPTC.2020.112934.

[22] A. C. Hindmarsh, "ODEPACK, A Systematized Collection of ODE Solvers," *IMACS Transactions on Scientific Computation*, vol. 1, pp. 55–64, 1983, Accessed: Feb. 18, 2025. [Online]. Available: https://computing.llnl.gov/sites/default/files/ODEPACK_pub1_u88007.pdf

[23] L. Petzold, "Automatic Selection of Methods for Solving Stiff and Nonstiff Systems of Ordinary Differential Equations," *https://doi.org/10.1137/0904010*, vol. 4, no. 1, pp. 136–148, Jul. 2006, doi: 10.1137/0904010.

[24] R. D. Minard, P. G. Hatcher, R. C. Gourley, and C. N. Matthews, "Structural Investigations of Hydrogen Cyanide Polymers: New Insights Using TMAH Thermochemolysis/GC-MS," *Origins of life and evolution of the biosphere*, vol. 28, no. 4, pp. 461–473, 1998, doi: 10.1023/A:1006566125815.

[25] R. Santalucia *et al.*, "From gaseous HCN to nucleobases at the cosmic silicate dust surface: an experimental insight into the onset of prebiotic chemistry in space," *Phys. Chem. Chem. Phys*, vol. 24, p. 7224, 2022, doi: 10.1039/d1cp05407d.

[26] H. Wakamatsu, Y. Yamada, T. Saito, I. Kumashiro, and T. Takenishi, "Synthesis of Adenine by Oligomerization of Hydrogen Cyanide," *Journal of Organic Chemistry*, vol. 31, no. 6, pp. 2035–2036, Jun. 1966, doi: 10.1021/JO01344A545.

[27] Y. Yamada, I. Kumashiro, and T. Takenishi, "Synthesis of Adenine and 4,5-Dicyanoimidazole from Hydrogen Cyanide in Liquid Ammonia," *Journal of Organic Chemistry*, vol. 33, no. 2, pp. 642–647, 1968, doi: 10.1021/JO01266A036.

[28] J. Oró, "Synthesis of adenine from ammonium cyanide," *Biochem Biophys Res Commun*, vol. 2, no. 6, pp. 407–412, Jun. 1960, doi: 10.1016/0006-291X(60)90138-8.

[29] M. Levy, S. L. Miller, and J. Oró, "Production of Guanine from NH4CN Polymerizations," *J Mol Evol*, vol. 49, no. 2, pp. 165–168, 1999, doi: 10.1007/PL00006539.

[30] S. Miyakawa, H. J. Cleaves, and S. L. Miller, "The Cold Origin of Life: B. Implications Based on Pyrimidines and Purines Produced From Frozen Ammonium Cyanide Solutions," *Origins of life and evolution of the biosphere*, vol. 32, no. 3, pp. 209–218, Jun. 2002, doi: 10.1023/A:1019514022822.

[31] J. P. Ferris, P. C. Joshi, E. H. Edelson, and J. G. Lawless, "HCN: A plausible source of purines, pyrimidines and amino acids on the primitive earth," *J Mol Evol*, vol. 11, no. 4, pp. 293–311, Oct. 1978, doi: 10.1007/BF01733839.

[32] M. Levy, S. L. Miller, K. Brinton, and J. L. Bada, "Prebiotic Synthesis of Adenine and Amino Acids Under Europa-like Conditions," *Icarus*, vol. 145, no. 2, pp. 609–613, Jun. 2000, doi: 10.1006/ICAR.2000.6365.

[33] A. W. Schwartz, H. Joosten, and A. B. Voet, "Prebiotic adenine synthesis via HCN oligomerization in ice," *Biosystems*, vol. 15, no. 3, pp. 191–193, Jun. 1982, doi: 10.1016/0303-2647(82)90003-X.
